# AMModels: An R package for storing models, data, and metadata to facilitate adaptive management

**DOI:** 10.1371/journal.pone.0188966

**Published:** 2018-02-28

**Authors:** Therese M. Donovan, Jonathan E. Katz

**Affiliations:** 1 U.S. Geological Survey, Vermont Cooperative Fish and Wildlife Research Unit, Rubenstein School of Environment and Natural Resources, University of Vermont, Burlington, Vermont, United States of America; 2 Vermont Cooperative Fish and Wildlife Research Unit, Rubenstein School of Environment and Natural Resources, University of Vermont, Burlington, Vermont, United States of America; Flinders University, AUSTRALIA

## Abstract

Agencies are increasingly called upon to implement their natural resource management programs within an adaptive management (AM) framework. This article provides the background and motivation for the R package, **AMModels**. AMModels was developed under R version 3.2.2. The overall goal of **AMModels** is simple: To codify knowledge in the form of models and to store it, along with models generated from numerous analyses and datasets that may come our way, so that it can be used or recalled in the future. **AMModels** facilitates this process by storing all models and datasets in a single object that can be saved to an .RData file and routinely augmented to track changes in knowledge through time. Through this process, **AMModels** allows the capture, development, sharing, and use of knowledge that may help organizations achieve their mission. While **AMModels** was designed to facilitate adaptive management, its utility is far more general. Many R packages exist for creating and summarizing models, but to our knowledge, **AMModels** is the only package dedicated not to the mechanics of analysis but to organizing analysis inputs, analysis outputs, and preserving descriptive metadata. We anticipate that this package will assist users hoping to preserve the key elements of an analysis so they may be more confidently revisited at a later date.

## Introduction and motivation

The R package, **AMModels** [1], is a tool for storing models, data, and metadata to facilitate adaptive management. Agencies are increasingly called upon to implement their natural resource management programs within an adaptive management (AM) framework [2–5]. Adaptive management is a key initiative for the U.S. Department of Interior, which offers the following definition [6]:

"Adaptive management promotes flexible decision making that can be adjusted in the face of uncertainties as outcomes from management actions and other events become better understood. Careful monitoring of these outcomes both advances scientific understanding and helps adjust policies or operations as part of an iterative learning process. Adaptive management also recognizes the importance of natural variability in contributing to ecological resilience and productivity. It is not a 'trial and error' process, but rather emphasizes learning while doing. Adaptive management does not represent an end in itself, but rather a means to more effective decisions and enhanced benefits. Its true measure is in how well it helps meet environmental, social, and economic goals, increases scientific knowledge, and reduces tensions among stakeholders."

Williams and Brown [6] explain that this definition emphasizes three key AM principles:

1. Uncertainty about management impacts
2. Iterative learning to reduce uncertainty
3. Improved management as a result of learning.

**Fig 1:**
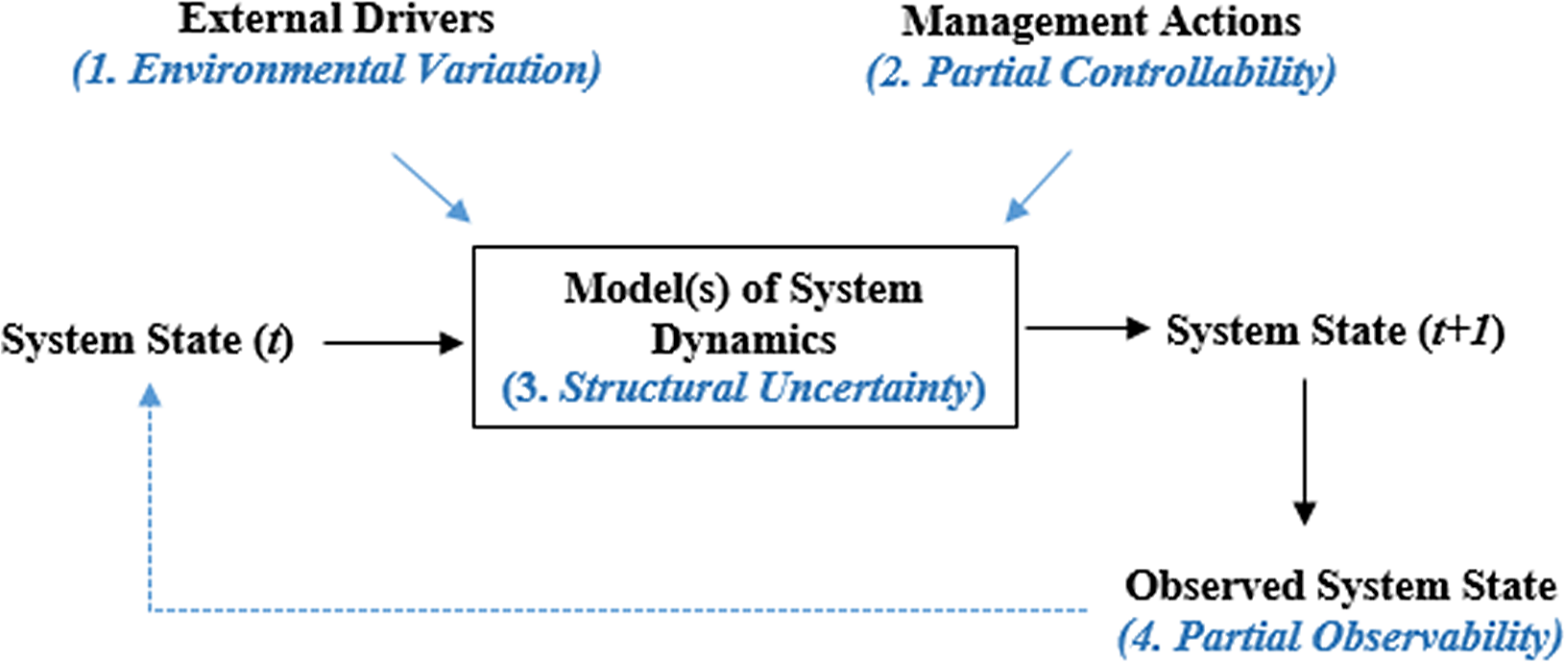
Components of an adaptive management system. Adapted from: National Conservation Training Center ALC3176; Nichols, Runge, and Johnson.

Models are a crucial part of this process. Natural resource managers are concerned with understanding the dynamics of a system of interest. The system state at time *t* is influenced by external drivers and management actions, which yield the state of the system at time *t + 1* (Fig 1).

At the heart of this diagram are **models**, which represent our current understanding of how a system of interest works. If a system is perfectly understood, a model can perfectly predict the state of the system at time *t* + 1, given the state at time *t* and the environmental and management forces that act on the system. However, environmental systems are rarely, if ever, understood with certainty. Sources of uncertainty that are highlighted in Fig 1 include:

1. Environmental Variation (also called aleatory uncertainty)—environmental factors that have inherent natural variability, such as rainfall and temperature, that affect the system state. Environmental variation can be better understood by additional study, but additional study cannot reduce it.
2. Partial Controllability—the uncertainty about how management actions will affect the system.
3. Structural Uncertainty—uncertainty with the model itself. This may arise when the model does not adequately reflect the system dynamics and/or when the parameters within a model are not well understood.
4. Partial Observability—uncertainty that arises when humans cannot observe the system state perfectly. This, in turn, may contribute to structural uncertainty.

The latter three sources of variation can be reduced by scientific study, and reducing uncertainty may lead to better decision making.

In an idealized adaptive management framework, the state of the system is sufficiently monitored to detect changes of interest. When that state appears to be out of sync with an agency's objectives, the agency considers a suite of possible management actions that can move the system towards its intended target. The model, though imperfect, is then used to help predict how each alternative may affect the state, pointing to the action(s) that best achieve agency goals. The selected management action(s), together with environmental drivers, then move the system to system state *t + 1*, and the process reiterates. A central premise of adaptive management is that the model of system dynamics can be continually improved with each passing iteration. Thus, the model of system dynamics changes as our understanding of the system improves [3].

Although the term adaptive management is liberally used in resource management, a large gap between the theory and the practice of adaptive management can exist [7–13]. Not all natural resource management problems fall within the adaptive management umbrella, and elucidating shared goals and objectives among stakeholders can be extremely challenging [14,15]. Even if objectives are well defined, in practice it can be difficult to understand how to implement an adaptive management program that seamlessly integrates data collection, models, and analysis. To aid at least part of this process, we developed the R package, **AMModels**, as a vehicle for organizing data, models, and metadata using the open source modeling platform, R [16]. R can be downloaded from the Comprehensive R Archive Network website (https://cran.r-project.org/).

## Objectives

This paper outlines the R package **AMModels**, which is foundational to our other AM packages [17,18]. The overall goal of **AMModels** is simple: To codify knowledge in the form of models and to store it, along with models generated from numerous analyses and datasets that may come our way, so that it can be used for future purposes. **AMModels** facilitates this process by storing all models and datasets in a single object that can be saved to an .RData file and routinely augmented to track changes in knowledge through time.

We begin by orienting the reader to R models and model outputs, which can be highly variable from package to package and function to function. We illustrate 3 modeling approaches. First, we analyze plant weight data with the function lm, which produces a linear model whose output is stored in an S3 object of class **lm**. Second, we analyze occupancy data of chorus frogs, which produces an occupancy model whose output is stored in an S4 object of class **unmarkedFitOccu**. Finally, we analyze fictitious apple data using a Bayesian analysis, in which the model inputs and outputs are not stored in an object with an analysis-specific class. In these examples, we do not dwell on the methods involved but rather focus on the structure of the analytical inputs and output. With this background, we then introduce the core functionality within the package, **AMModels**, including a Shiny application called **AM Model Manager** which enables the use of these functions in user-friendly format.

## Models in R

Models come in a rich variety of flavors, but a common flavor is a model that is generated from a statistical analysis.

### Example 1: Linear models

As an example of a model generated from previous analysis, consider the code from the lm helpfile featuring plant weights as a function of a treatment. We use this example generically; in the context of adaptive management, a resource manager may be interested in how a treatment or management action affects a resource of interest (e.g., plant weight). Here, a linear model is fit to the observed data, where weight is predicted by the treatment effect (group).

**require**(graphics)

*# Annette Dobson (1990) 'An Introduction to Generalized Linear Models'*.

*# Page 9*: *Plant Weight Data*.

ctl <- **c**(4.17, 5.58, 5.18, 6.11, 4.5, 4.61, 5.17, 4.53, 5.33, 5.14)

trt <- **c**(4.81, 4.17, 4.41, 3.59, 5.87, 3.83, 6.03, 4.89, 4.32, 4.69)

group <- **gl**(n = 2, k = 10, length = 20, labels = **c**("Ctl", "Trt"))

weight <- **c**(ctl, trt)

*# store the data within a dataframe*

data <- **data.frame**(weight, group)

*# run the analysis*

lm.D9 <- **lm**(weight ~ group, data = data)

*# look at the class of lm*.*D9*

**class**(lm.D9)

[1] "lm"

Model results are stored in an object of class **lm**, which is structured as a list with 13 elements. This can be verified with the str function. A quick way to see hints about the contents of this object is to use the names function.

*# look at the names of the lm components*

**names**(lm.D9)

$$\begin{array}{ccccc} \left[1\right] & "\mathrm{coefficients}" & "\mathrm{residuals}" & "\mathrm{effects}" & "\mathrm{rank}"\\ \left[5\right] & "\mathrm{fitted}.\mathrm{values}" & "\mathrm{assign}" & "\mathrm{qr}" & "\mathrm{df}.\mathrm{residual}"\\ \left[9\right] & "\mathrm{contrasts}"\; & "\mathrm{xlevels}" & "\mathrm{call}" & \;"\mathrm{terms}"\\ \left[13\right] & "\mathrm{model}"\; \end{array}$$

Typically, analytical outputs contain not only the model results, but additional information regarding the analysis. For example, the data that were input to the analysis are stored in the list item named **model**.

*# extract the analysis inputs (data)*

lm.D9["model"]

$model

$$\begin{array}{ccc} & \mathrm{weight} & \mathrm{group}\\ 1 & 4.17 & \mathrm{Ctl}\\ 2 & 5.58 & \mathrm{Ctl}\\ 3 & 5.18 & \mathrm{Ctl}\\ 4 & 6.11 & \mathrm{Ctl}\\ 5 & 4.50 & \mathrm{Ctl}\\ 6 & 4.61 & \mathrm{Ctl}\\ 7 & 5.17 & \mathrm{Ctl}\\ 8 & 4.53 & \mathrm{Ctl}\\ 9 & 5.33 & \mathrm{Ctl}\\ 10 & 5.14 & \mathrm{Ctl}\\ 11 & 4.81 & \mathrm{Trt}\\ 12 & 4.17 & \mathrm{Trt}\\ 13 & 4.41 & \mathrm{Trt}\\ 14 & 3.59 & \mathrm{Trt}\\ 15 & 5.87 & \mathrm{Trt}\\ 16 & 3.83 & \mathrm{Trt}\\ 17 & 6.03 & \mathrm{Trt}\\ 18 & 4.89 & \mathrm{Trt}\\ 19 & 4.32 & \mathrm{Trt}\\ 20 & 4.69 & \mathrm{Trt} \end{array}$$

It is generally unnecessary to use such list indexing to work with the lm output, as the authors of the lm function provide numerous functions (methods) for extracting and manipulating content stored in an object of class **lm**. The names of the methods that work on a particular class can be displayed with the methods function:

*# return the functions that operate on an object with class lm*

**methods**(class = "lm")

$$\begin{array}{ccccc} \left[1\right] & \mathrm{add}1 & \mathrm{alias} & \mathrm{anova} & \mathrm{case}.\mathrm{names}\\ \left[5\right] & \mathrm{coerce} & \mathrm{confint} & \mathrm{cooks}.\mathrm{distance} & \mathrm{deviance}\\ \left[9\right] & \mathrm{dfbeta} & \mathrm{dfbetas} & \mathrm{drop}1 & \mathrm{dummy}.\mathrm{coef}\\ \left[13\right] & \mathrm{effects} & \mathrm{extractAIC} & \mathrm{family} & \mathrm{formula}\\ \left[17\right] & \mathrm{hatvalues} & \mathrm{influence} & \mathrm{initialize} & \mathrm{kappa}\\ \left[21\right] & \mathrm{labels} & \mathrm{logLik} & \mathrm{model}.\mathrm{frame} & \mathrm{model}.\mathrm{matrix}\\ \left[25\right] & \mathrm{nobs} & \mathrm{plot} & \mathrm{predict} & \mathrm{print}\\ \left[29\right] & \mathrm{proj} & \mathrm{qr} & \mathrm{residuals} & \mathrm{rstandard}\\ \left[33\right] & \mathrm{rstudent} & \mathrm{show} & \mathrm{simulate} & \mathrm{slotsFromS}3\\ \left[37\right] & \mathrm{summary} & \mathrm{variable}.\mathrm{names} & \mathrm{vcov} \end{array}$$

see '?methods' for accessing help and source code

For instance, the residuals method will extract the "residuals," the predict method will extract the "fitted.values" from an **lm** object, the model.frame method will return the data input, and the summary method will print the model residuals, coefficients and their associated statistics.

**summary**(lm.D9)

Call:

lm(formula = weight ~ group, data = data)

Residuals:

$$\begin{array}{ccccc} \;\mathrm{Min} & \;1Q & \mathrm{Median} & \;3Q & \;\mathrm{Max}\\ -1.0710 & -0.4938 & 0.0685 & 0.2462 & 1.3690 \end{array}$$

Coefficients:

$$\begin{array}{cccccc} & \mathrm{Estimate}\mathrm{Std}. & \mathrm{Error} & t\;\mathrm{value} & \;\mathrm{Pr}(>|t|) & \\ \left(\mathrm{Intercept}\right) & 5.0320 & 0.2202 & 22.850 & 9.55e-15 & ***\\ \mathrm{groupTrt}\; & -0.3710 & 0.3114 & -1.191 & \;0.249 & \end{array}$$

Signif. codes:      0 '***' 0.001 '**' 0.01 '*' 0.05 '.' 0.1 ' ' 1

Residual standard error: 0.6964 on 18 degrees of freedom

Multiple R-squared:      0.07308,        Adjusted R-squared:      0.02158

F-statistic: 1.419 on 1 and 18 DF,      p-value: 0.249

The output of the **lm** function can be expressed as a basic linear model with the form:

$$Y_{i}=\beta_{0}+\beta_{1}*X_{i}+\epsilon_{i}.$$

We can substitute in our variable names and coefficients:

$${weight}_{i}=5.0320-0.3710*{treatment}_{i}.$$

If a resource manager is interested in a system state (plant weight at time *t*), this model can be used to help determine if a management action (the treatment) should be used to shift the system to a new state at time *t + 1*. Average plant weight for the control group was 5.0320 units, while average plant weight for the treatment group was 5.0320–0.3710 = 4.661. These averages are not far apart, and the model results indicate that the treatment may not yield a strong change in plant weight.

An important part of the lm output is the model 'fit,' which conveys how well the linear model fits the observed data. In this case, the model does not fit very well (adjusted R-squared = 0.02158). Additionally, the standard errors associated with each coefficient reflect our uncertainty in the parameter estimates, and thus influence our belief that the treatment does not strongly affect plant weight. Both represent a form of structural uncertainty in the model. And because treatment, a management action, is included within this model, partial controllability is another source of uncertainty.

To summarize this example, the analytical inputs are stored as a dataframe, and the analytical outputs are returned as an object of class **lm**.

### Example 2. Occupancy modeling

Model uncertainty may also result from the structural form of the model itself. To illustrate, we now consider two alternative models from a single season occupancy analysis for a wildlife species of interest [19]. The primary input for a single season occupancy analysis consists of raw survey data across multiple study sites in the form of detection and non-detection data, where 1 means the species was detected on a given survey at a given site and 0 means the species was not detected. Additional inputs include covariates associated with each site (such as habitat type or elevation) and covariates associated with the conditions associated with each survey (such as date, time, weather conditions, or observer). The analysis returns maximum likelihood estimates for occupancy (the probability that a site is occupied) and detection (the probability that a species will be detected, given it is present on a site).

The single season occupancy analysis can be run with the occu function in the R package, unmarked [20]. Here, we use the code in the occu helpfile to run a single-season occupancy analysis on chorus frog (*Pseudacris feriarum*) occupancy at 130 sites in Delaware [21].

*# load package unmarked*

**suppressPackageStartupMessages**(**library**(unmarked))

*# load the chorus frog data*

**data**(frogs)

*# create an unmarked frame*

pferUMF <- **unmarkedFrameOccu**(pfer.bin)

*# get the number of sites in which frogs were surveyed*

num.sites <- **numSites**(pferUMF)

*# add a fake site level covariate called sitevar1 for illustration*

*# specify a seed for a random number generator; used for reproducibility*.

**set.seed**(20)

site.sums <- **rowSums**(pferUMF@y, na.rm = T)

sitevar1 = **round**(**rnorm**(n = num.sites, mean = **ifelse**(test = site.sums > 0, yes = 15, no = 10), sd = 3), 2)

**siteCovs**(pferUMF) <- **data.frame**(sitevar1)

*# add a fake observation covariate called obsvar1 for illustration*

**obsCovs**(pferUMF) <- **data.frame**(obsvar1 = **c**(**rep**(1:3, num.sites)))

*# look at the first 10 records of the resulting unmarkedFrame object*.

**head**(pferUMF, n = 10)

Data frame representation of unmarkedFrame object.

$$\begin{array}{cccccccc} & y.1 & y.2 & y.3 & \mathrm{sitevar}1 & \mathrm{obsvar}1.1 & \mathrm{obsvar}1.2 & \mathrm{obsvar}1.3\\ 1 & 1 & 0 & \mathrm{NA} & 18.49 & 1 & 2 & 3\\ 2 & 1 & 0 & 0 & 13.24 & 1 & 2 & 3\\ 3 & 1 & 0 & 0 & 20.36 & 1 & 2 & 3\\ 4 & 1 & 0 & 0 & 11.00 & 1 & 2 & 3\\ 5 & 0 & 0 & 0 & 8.66 & 1 & 2 & 3\\ 6 & 0 & 0 & 0 & 11.71 & 1 & 2 & 3\\ 7 & 0 & 0 & 0 & 1.33 & 1 & 2 & 3\\ 8 & 0 & 0 & 0 & 7.39 & 1 & 2 & 3\\ 9 & 1 & 0 & 0 & 13.61 & 1 & 2 & 3\\ 10 & 0 & 0 & 0 & 8.33 & 1 & 2 & 3 \end{array}$$

The **unmarkedFrame** object is the input needed to run a single season occupancy analysis with the occu function. In the (human-readable) data frame representation displayed above, the first three columns (labeled y.1, y.2, and y.3) give the results of the chorus frog surveys, of which there are three. For instance, on site 1, chorus frogs were detected on survey 1, not detected on survey 2, and survey 3 was not conducted, yielding 1, 0, NA in row one, columns one through three of the dataset. On site 2, chorus frogs were detected on survey 1, not detected on survey 2, and not detected on survey 3, yielding 1, 0, 0 in row two.

There is a single site-level covariate in this dataset, named **sitevar1**, which was simulated such that sites with detections generally had higher **sitevar1** values compared to sites with no detections. For example, site 1's **sitevar1** value is 18.49, while site 6's **sitevar1** value is 11.71.

There is a single detection covariate in this dataset, named **obsvar1**. Notice that there are three columns associated with **obsvar1**, representing the value of **obsvar1** for each of the three surveys. Here, **obsvar1**'s value is the survey number (1, 2, or 3), which presumably ranks the survey date.

The occu function can be used to analyze the **unmarkedFrame** object with a single-season occupancy model. This function requires that two models are specified as a double right-hand side formula: one component for detection (which is specified first) and one for occupancy. Here, we run the occupancy model when detection is a function of **obsvar1** and occupancy is a function of **sitevar1**, and point to the **unmarkedFrame** called **pferUMV** as the required data input.

*# fit a single-season occupancy model where detection is a function of obsvar1 and occupancy is a function of sitevar1*

fm1 <- **occu**(formula = ~obsvar1 ~ sitevar1, data = pferUMF)

The output of this analysis, named here as **fm1**, is an object is of class **unmarkedFitOccu**, which we can verify with the class function. The "package" attribute identifies the R package that produced this object, which will be handy to jog our human memory when we store models for future re-use.

*# get the class of fm1*

**class**(fm1)

[1] "unmarkedFitOccu"

attr(,"package")

[1] "unmarked"

The output contains the raw information needed to draw conclusions about chorus frog occupancy rates and the covariates that (may) affect occupancy state and detection. The output is stored in 13 slots, and includes not only the model output, but also the details about the analysis, including the two primary inputs (formula and data).

*# get the slots names associated with fm1 output*

**slotNames**(fm1)

$$\begin{array}{cccc} \left[1\right] & "\mathrm{knownOcc}" & "\mathrm{fitType}" & "\mathrm{call}"\\ \left[4\right] & "\mathrm{formula}" & "\mathrm{data}" & "\mathrm{sitesRemoved}"\\ \left[7\right] & "\mathrm{estimates}" & "\mathrm{AIC}" & "\mathrm{opt}"\\ \left[10\right] & "\mathrm{negLogLike}" & "\mathrm{nllFun}" & "\mathrm{bootstrapSamples}"\\ \left[13\right] & "\mathrm{covMatBS}" \end{array}$$

As with the lm output, the authors of **unmarked** provide numerous methods for summarizing and extracting results from an **unmarkedFitOccu** object. For example, the summary method will return the model coefficients, the AIC, and other information for evaluating the model.

*# run unmarked's summary function*

**summary**(fm1)

Call:

occu(formula = ~obsvar1 ~ sitevar1, data = pferUMF)

Occupancy (logit-scale):

$$\begin{array}{ccccc} & \mathrm{Estimate} & \mathrm{SE} & z & P(>|z|)\\ \left(\mathrm{Intercept}\right) & -7.340 & 1.480 & -4.96 & 7.08e-07\\ \mathrm{sitevar}1\; & 0.575 & 0.124 & 4.63 & 3.71e-06 \end{array}$$

Detection (logit-scale):

$$\begin{array}{ccccc} & \mathrm{Estimate} & \mathrm{SE} & z & P(>|z|)\\ \left(\mathrm{Intercept}\right) & 4.71 & 1.083 & 4.35 & 1.38e-05\\ \mathrm{obsvar}1\; & -3.57 & 0.665 & -5.37 & 8.06e-08 \end{array}$$

AIC: 159.3813

Number of sites: 130

optim convergence code: 0

optim iterations: 38

Bootstrap iterations: 0

The occu function models on the log-odds (logit) scale, which is required because occupancy and detection rates are probabilities that are constrained between 0 and 1. Thus, the coefficients of an analysis output are on the logit scale. The chorus frog occupancy model has the form:

$${logit(occupancy)}_{i}=-7.340+0.575*{sitevar1}_{i}$$

*# create a matrix with a range of sitecov values*

data <- **cbind**(1, **seq**(from = 0, to = 30, length.out = 100))

*# calculate the linear score (logit) across a range of sitecov values*

lc <- **linearComb**(obj = fm1["state"], data)

*# use unmarked's backTransform function to convert the linear score (logit)*

*# to probability*

btlc <- **backTransform**(lc)

*# convert to dataframe and add upper and lower confidence intervals*

btlc <- **as**(btlc, "data.frame")

btlc$uci <- btlc$Estimate + 1.96 * btlc$SE

btlc$lci <- btlc$Estimate—1.96 * btlc$SE

*# plot the predicted occupancy rates*

**par**(family = "serif")

**plot**(x = btlc$sitevar1, y = btlc$Estimate, type = "l", main = "Chorus Frog Occupancy as a Function of Sitevar1",

xlab = "Value of sitevar1", ylab = "Predicted Occupancy")

*# add uncertainty in the form of confidence interval lines*

**lines**(x = btlc$sitevar1, y = btlc$lci, lty = "dashed", col = "gray")

**lines**(x = btlc$sitevar1, y = btlc$uci, lty = "dashed", col = "gray")

This model (if it is shown to be adequate) can be of great utility to natural resource managers interested in managing chorus frogs. The model of chorus frog system occupancy suggests that sites with **sitevar1** levels > ~ 15 units have a high chance of being occupied. If **sitevar1** can be manipulated by management activities, this model may allow resource managers to predict how the state of the system (occupancy) at time *t* can be shifted to a desired state at time *t + 1* (Fig 1) by manipulating **sitevar1** across sites.

**Fig 2:**
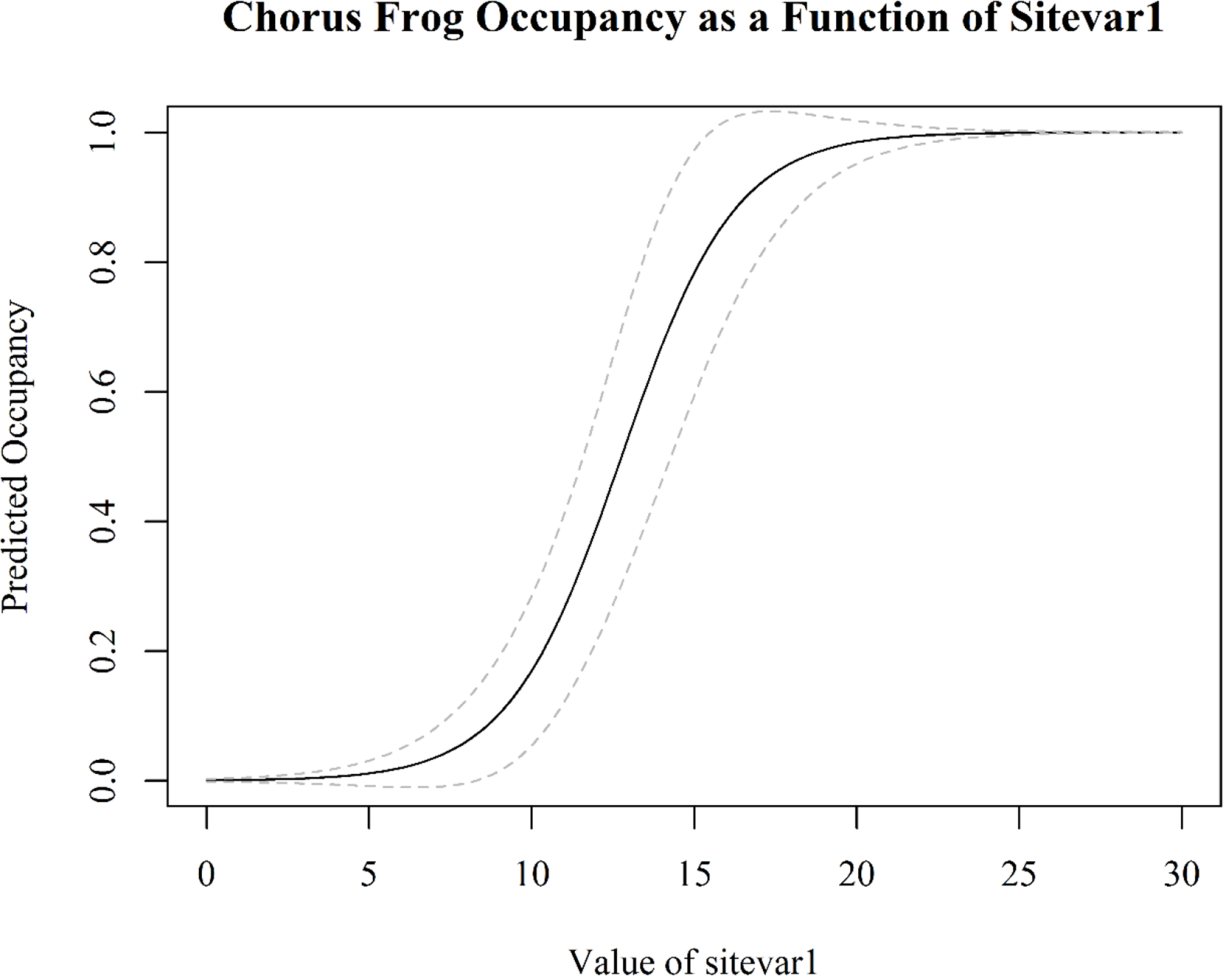
Chorus frog occupancy as a function of the simulated covariate, sitevar1.

We can back-transform the logit score to a probability with the log-odds transformation, and use the code on the following page to plot our results (Fig 2).

The occu outputs also contain a detection model, which, based on the model output, now has the form:

$${logit(detection)}_{i}=4.71+-3.57*{obsvar1}_{i}$$

This model (if it is shown to be adequate) suggests that as **obsvar1** increases, the detection probability of chorus frogs decreases (a scan of the raw data shows that most frogs were detected in survey 1 of the study). This model attempts to deal with partial observability in Fig 1. That is, the analyst attempts to discover those covariates that affect observability so that this source of uncertainty can be reduced. The occu model results show that conditions associated with first chorus frog surveys resulted in much higher detection probability than in later surveys. Although this result will probably not help identify management actions that push the system state (occupancy), this information can be used to improve the accuracy of the monitoring program, and hence reduce partial observability (Fig 1). This, in turn, may lead to a better model of system dynamics.

In most cases, multiple models are evaluated and compared to determine which model(s) best explain the observed, raw data [22]. To wit, we now run a second occupancy analysis on the chorus frog data, in which both detection and occupancy have no covariates (i.e., only the intercepts will be estimated):

*# fit a single season occupancy model with no covariates*

fm2 <- **occu**(formula = ~1 ~ 1, data = pferUMF)

We can now compare the two models with the fitList and modSel functions in **unmarked**.

*# create a list of models that will be compared*

fl <- **fitList**(fm1. = fm1, fm2. = fm2)

*# perform model selection analysis*

ms <- **modSel**(object = fl, nullmod = "fm2.")

*# view the results of the analysis*

ms

$$\begin{array}{ccccccc} & \mathrm{nPars} & \;\mathrm{AIC} & \;\mathrm{delta} & \mathrm{AICwt} & \mathrm{cumltvWt} & \;\mathrm{Rsq}\\ \mathrm{fm}1. & \;4 & 159.38 & \;0.00 & 1.0e+00 & \;1.00 & 0.65\\ \mathrm{fm}2. & \;2 & 261.33 & 101.95 & 7.3e-23 & \;1.00 & 0.00 \end{array}$$

Without getting into the weeds [22], the AIC weights (AICwt) from this analysis suggest that model 1 (**fm1**) has 100% support, meaning that the model with no covariates (**fm2**) has 0 support. In other words, the structure of **fm2** has so much structural uncertainty compared to **fm1** that it serves no purpose in chorus frog management. We would still like some evidence that **fm1** is a good 'fit' to our observed field data, which we can do with the MacKenzie and Bailey Goodness-of-fit Test [19]. In R, this test is implemented by the mb.gof.test function in the package **AICcmodavg** [23], typically executed with 1000’s of trials.

*# load the package AICcmodavg*

**library**(AICcmodavg)

*# run the goodness of fit test*

AICcmodavg::**mb.gof.test**(mod = fm1, nsim = 100, plot.hist = FALSE)

MacKenzie and Bailey goodness-of-fit for single-season occupancy model

Pearson chi-square table:

$$\begin{array}{ccccc} & \mathrm{Cohort} & \mathrm{Observed} & \mathrm{Expected} & \mathrm{Chi}-\mathrm{square}\\ 000 & 0 & 58 & 66.22 & 1.02\\ 010 & 0 & 4 & 0.77 & 13.43\\ 100 & 0 & 35 & 27.49 & 2.05\\ 00\mathrm{NA} & 1 & 12 & 9.19 & 0.86\\ 10\mathrm{NA} & 1 & 1 & 3.41 & 1.70\\ 0\mathrm{NANA} & 2 & 17 & 14.58 & 0.40\\ 1\mathrm{NANA} & 2 & 3 & 5.42 & 1.08 \end{array}$$

Chi-square statistic = 23.4566

Number of bootstrap samples = 100

P-value = 0.04

Quantiles of bootstrapped statistics:

$$\begin{array}{ccccc} 0\% & 25\% & 50\% & 75\% & 100\%\\ 0.22 & 2.13 & 3.26 & 6.00 & 74.71 \end{array}$$

Estimate of c-hat = 3.99

Again, without dwelling on details, this analysis suggests that even **fm1** does not adequately explain the observed data (with a very high overall Chi-square statistic of ~ 23, meaning our observed and expected results are not very well matched). Keep in mind that we simulated the covariates in this model, so a lack of fit is not surprising. However, if the data were not simulated, this goodness of fit test suggests that **fm1** has significant structural uncertainty, and we should look for a better occupancy model, or even a different modeling approach.

To summarize this example, the analysis inputs are stored in an object of class **unmarkedFrame**, and the analysis outputs are returned as an object of class **unmarkedFitOccu**.

### Example 3. Bayesian modeling

Our final example is a Bayesian approach, which is an ideal modeling framework for adaptive management because it codifies learning through time. The International Society of Bayesian Analysis describe the Bayesian paradigm, which can be summarized as follows. First, current knowledge about the model parameters of interest is expressed by placing a 'prior probability distribution' on the parameters, which is often written as

p(θ).

Second, when new data *y* become available, the likelihood of observing the data given the model parameters is calculated, which is often expressed as

p(y|θ).

Finally, the likelihood is then combined with the prior to produce an updated probability distribution called the 'posterior distribution,' upon which all Bayesian inference is based. The posterior distribution is often written as:

p(θ|y).

Bayes' Theorem is an elementary identity in probability theory, and states how the prior distribution is updated to the posterior distribution in light of new information, *y*. More precisely,

$$p(\theta |y)=\frac{p(\theta )p(y|\theta )}{\int p(\theta )p(y|\theta )d\theta }.$$

Normally a Bayesian analysis in R would be conducted via a package such as rjags [24], in which the analytical inputs are stored in an object of class **jags** and the model output is stored in an object of class **mcmc.list**. Here, though, to illustrate the complete flexibility of **AMModels**, we will store inputs and outputs as typical S3 objects.

**Fig 3:**
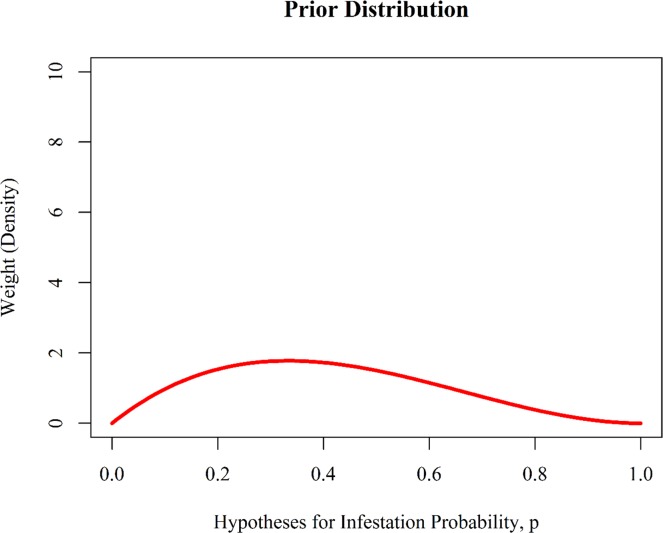
The prior distribution for infestation rate, p, is a beta distribution with *α* = 2 and *β* = 3.

Suppose we have a need to estimate the probability of that apples in an orchard will be infested by the apple maggot. Let's call this probability *p*. With a Bayesian analysis, the analyst must provide **prior** distributions for each parameter of interest, and we are focused on the single parameter, *p*. We can represent our beliefs in alternative values of *p* with a beta distribution—a probability density function that is controlled by two parameters, normally called alpha (*α*) and beta (*β*). Let's first graph our prior distribution for the unknown parameter, *p* (Fig 3):

*# establish prior distribution for the unknown parameter*, *p (a beta*

*# distribution)*

alpha.prior <- 2

beta.prior <- 3

p <- **seq**(from = 0, to = 1, by = 0.01)

prior.density <- **dbeta**(p, shape1 = alpha.prior, shape2 = beta.prior, ncp = 0,

log = FALSE)

*# graph the prior distribution*

**par**(family = "serif")

**plot**(x = p, y = prior.density, type = "l", ylim = **c**(0, 10), ylab = "Weight (Density)", xlab = "Hypotheses for Infestation Probability, p", col = "red", lwd = 3, main = "Prior Distribution")

Our model for *p* is fully represented by a beta distribution with parameters *α* = 2 and *β* = 3. This particular model suggests that there is quite a bit of uncertainty regarding *p*.

In an adaptive management framework, the goal is to reduce this uncertainty. For example, if we collect new data on infestation rates, we can use Bayes' Theorem and update our beliefs in alternative hypotheses for *p*. Suppose we collect a sample of 100 apples from our orchard, and that 25 of them are infested. We can now combine this new data, along with the parameters of the prior distribution for *p*, to generate a posterior distribution that reflects our updated knowledge regarding infestation probability. For this particular problem, the parameters for this posterior distribution can be calculated quickly with the following equations, where *n* is the total number of apples in our sample, and *y* is the total number of infected apples:

- *α*_posterior_ = *α*_prior_ + *y* = 2 + 25 = 27
- *β*_posterior_ = *β*_prior_ + *n* − *y* = 3 + 100–25 = 78

**Fig 4:**
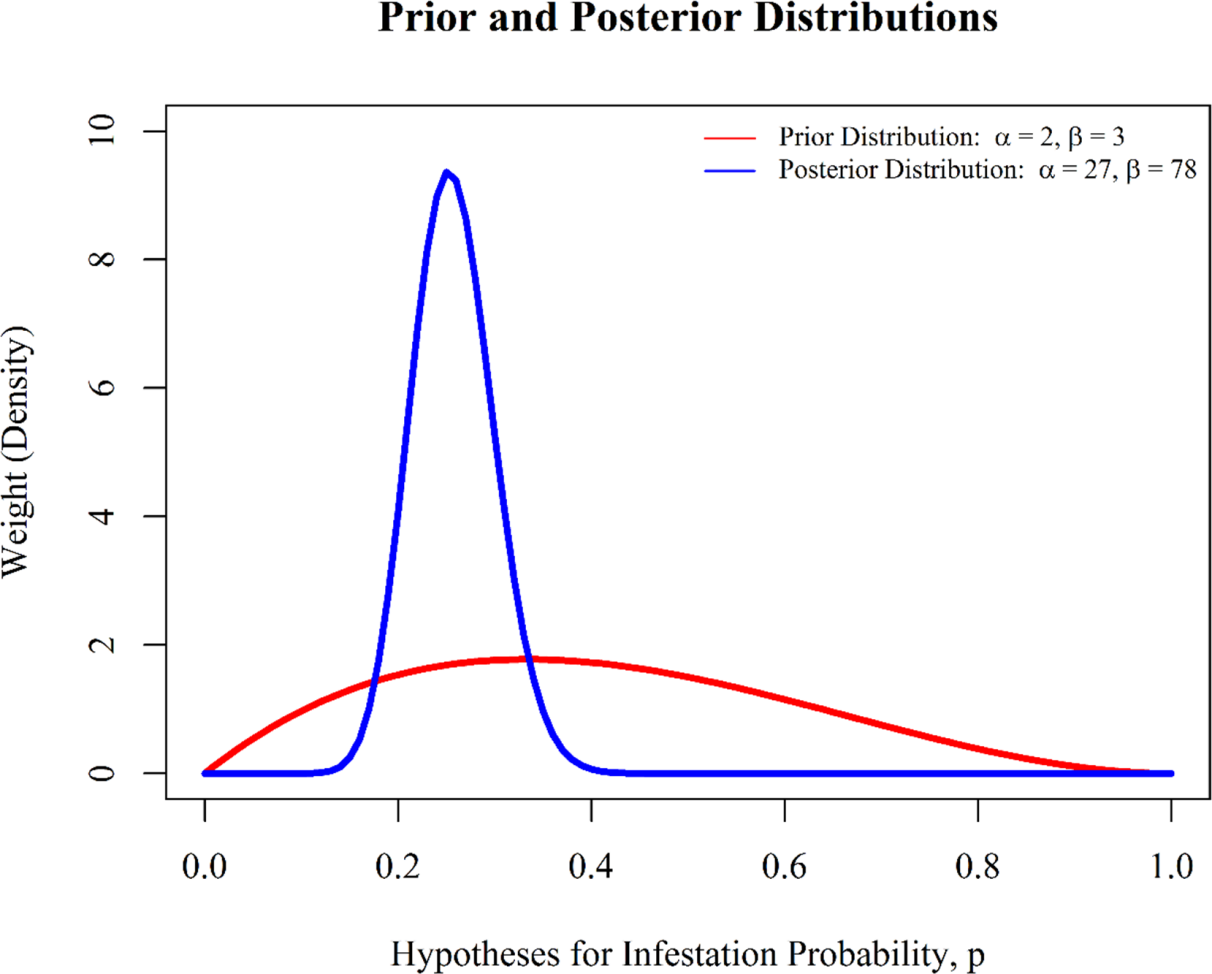
The prior and posterior distributions for infestation probability, p.

A graph of the prior distribution and the posterior distribution can be displayed in Fig 4 using the code below: (Fig 4).

*# establish posterior distribution for the unknown parameter*, *p (a beta*

*# distribution)*

alpha.posterior <- 27

beta.posterior <- 78

*# calculate the densities associated with the updated distribution*

posterior.density <- **dbeta**(p, shape1 = alpha.posterior, shape2 = beta.posterior, ncp = 0, log = FALSE)

*# plot the prior distribution*

**par**(family = "serif")

**plot**(x = p, y = prior.density, xlim = **c**(0, 1), ylim = **c**(0, 10), type = "l",

ylab = "Weight (Density)", xlab = "Hypotheses for Infestation Probability, p", main = "Prior and Posterior Distributions", col = "red", lwd = 3)

*# add the posterior distribution*

**lines**(x = p, y = posterior.density, col = "blue", lwd = 3)

*# add legend*

**legend**("topright", legend = **c**(**expression**(**paste**("Prior Distribution: ", alpha, " = 2, ", beta, " = 3")), **expression**(**paste**("Posterior Distribution: ",

alpha, " = 27, ", beta, " = 78"))), lty = 1, col = **c**("red", "blue"), bty = "n", cex = 0.75)

Notice how the new data has yielded updated knowledge about infestation probability, considerably reducing our uncertainty. If you were to continue collecting data, you would now use the parameters from the posterior distribution as the prior distribution, combine it with another set of new data, and then generate a third, updated model for apple infestation. These are valuable models that reflect the current knowledge of apple infestation and through time, even though they do not link to management activities or to environmental drivers.

Unlike our previous two examples, the inputs and outputs for this analysis are not shaped or stored in dedicated objects such as unmarkedFitOccu. Here, to make the simple point that **AMModels** can store user-defined analysis inputs and outputs, we will store our apple analysis as a list, and link the second analysis with the first by specifying a list element named "prior" (note that, in this hypothetical example, **apple.m2** is connected with **apple.m1** via a list element named "prior").

*# create model1 as a list; coefficients*

apple.m1 <- **list**(name = "apple.m1", distribution = "beta", prior = **list**(alpha = 2, beta = 3))

*# create model2 as a list; coefficients*

apple.m2 <- **list**(name = "apple.m2", distribution = "beta", prior = "apple.m1", data = **c**(y = 25, n = 100), posterior = **list**(alpha = 27, beta = 78))

*# look at the structure apple*.*m1*

**str**(apple.m1)

List of 3

  $ name         : chr "apple.m1"

  $ distribution: chr "beta"

  $ prior        : List of 2

    ..$ alpha: num 2

    ..$ beta: num 3

*# look at the structure apple*.*m2*

**str**(apple.m2)

List of 5

  $ name         : chr "apple.m2"

  $ distribution: chr "beta"

  $ prior        : chr "apple.m1"

  $ data         : Named num [1:2] 25 100

    ..- attr(*, "names") = chr [1:2] "y" "n"

  $ posterior      : List of 2

    ..$ alpha: num 27

    ..$ beta   : num 78

## Models in Adaptive Management

An enormous variety of analytical approaches exist to meet a variety of needs (e.g., [25–31]). The three examples are intended to simply highlight a few key points with respect to analytical inputs and outputs in R in relation to adaptive management:

1. Analytical inputs to R functions are unique to the analytical framework (e.g. Example 1's input was a dataframe while Example 2's input was an **unmarkedFrame**).
2. Analytical outputs from R functions are also unique to the analytical framework (e.g., Example 1's output was class **lm** while Example 2's output was class **unmarkedFitOccu**).
3. Outputs that are models represent a state of knowledge.
4. All models include elements of uncertainty. In the first two examples provided, there is uncertainty in the coefficient estimates, and there is uncertainty in the model structure.
5. Models may be a primary output from an analysis, but they may also be an input for future analyses (e.g., Example 3).
6. Information associated with an analysis itself (i.e., the analysis metadata) can preserve the context of the analysis so that the analysis may be more confidently revisited at a later date.

In an adaptive management program, the models must be explicit, and models may be modified through time as more and more is learned about the system being managed. The notion of adaptive management is to acknowledge uncertainty at every turn, and reduce this uncertainty by careful monitoring and study over time.

## The R package, AMModels

The overall goal of **AMModels** is straight-forward: To codify knowledge in the form of models and to store it, along with models generated from numerous analyses and datasets that may come our way, so that it can be used in the future. **AMModels** facilitates this process by storing analysis inputs, outputs, and metadata in a single object that can be repeatedly updated to track changes in knowledge through time.

In this section, we demonstrate the core functionality and typical workflow of **AMModels**. Many of these examples are highlighted in the package’s helpfiles. In the final section of this paper, we introduce the **AM Model Manager**, a Shiny application [32] that simplifies the storage and retrieval of models and associated datasets.

*# install*.*packages("AMModels")*

*# load AMModels*

**library**(AMModels)

### amModelLib Object

A central idea of **AMModels** is that all models and datasets, along with their metadata, are stored in an **amModelLib** object, where "Lib" stands for "library." This is an S4 object that can be updated and used repeatedly as a way of carting around knowledge in the form of models. Think of the **amModelLib** as a vault or repository that stores datasets (analysis inputs) and models (analysis outputs). The function amModelLib creates the object.

*# create an amModelLib object*

mymodels <- **amModelLib**(description = "This AM Model Library stores models and data.", info = **list**(owner = "me", email = "me@somewhere.com"))

*# view the amModelLib*

mymodels

Description:

[1] This AM Model Library stores models and data.

Info:

    owner

      [1] me

    email

      [1] me@somewhere.com

    date.created

      [1] 2017-10-04 07:28:28

Models:

   --- There are no models ---

Data:

   --- There are no datasets ---

An **amModelLib** objects contain 4 slots, which can be verified with the slotNames function.

*# view the slot names*

**slotNames**(mymodels)

[1] "models"          "data"          "info"          "description"

**Fig 5:**
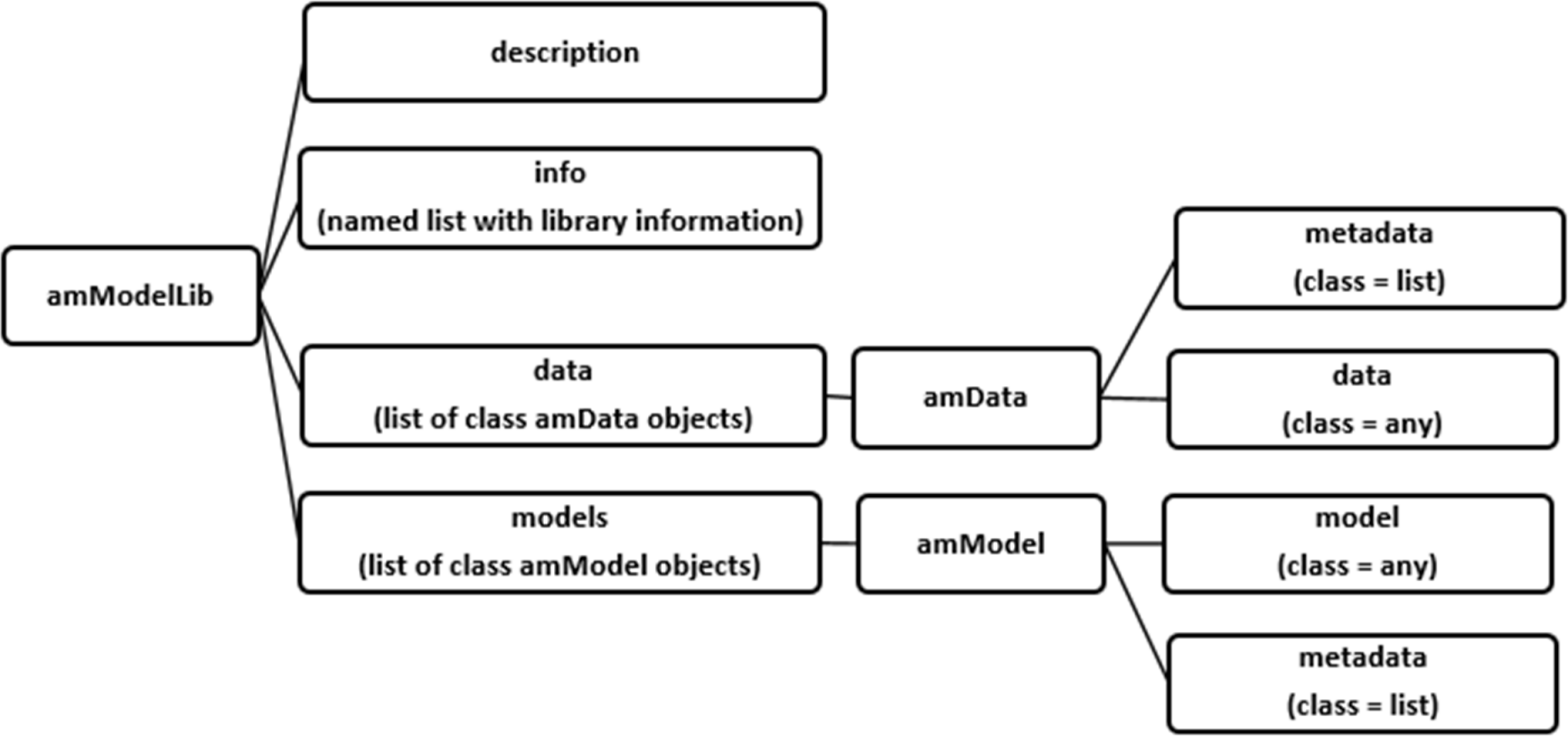
The structure of an amModelLib object, consisting of four slots.

Fig 5 highlights the four slots of the **amModelLib**:

1. **description**. A text field that describes the **amModelLib**. Generally the description is one sentence long. Additional information can be stored in the **info** slot.
2. **info**. A named list that contains additional information about the **amModelLib**, such as the name of the person who creates and maintains the **amModelLib**, and any additional related metadata pertaining to the library itself.
3. **data**. This slot stores datasets, i.e., analysis inputs. Each dataset has a class of **amData**, which consists of two slots: 1) datasets are stored in the data slot, and 2) metadata about the dataset are stored in the metadata slot.
4. **models**. This slot stores models, i.e., analysis outputs. Each model has a class of **amModel**, which consists of two slots: 1) models are stored in the model slot, and 2) metadata about the model are stored in the metadata slot.

Each slot is described below. The package **AMModels** contains functions to create the **amModelLib** object, functions to create objects of class **amModel** and **amData**, and methods to insert, extract, and delete items from the **amModelLib** object. A cheat sheet of the various functions can be found in Supplement 1.

#### The Description slot

The description slot can be viewed or updated with the function ammlDesc.

*# show the amModelLib description*

**ammlDesc**(amml = mymodels)

[1] "This AM Model Library stores models and data."

*# update the description*

**ammlDesc**(amml = mymodels) <- "This AM Model Library stores analysis inputs (data) and analysis outputs (models) associated with the AMModels package vignette."

*# show the amModelLib description*

**ammlDesc**(amml = mymodels)

[1] "This AM Model Library stores analysis inputs (data) and analysis outputs (models) associated with the AMModels package vignette."

#### The Info slot

The info slot of an **amModelLib** is a named list that stores information about the model library itself. This list is unlimited in length. Information is stored in name (key) = value syntax, such as:

- owner = "me"
- email = "me@somewhere.com"

The **amModelLib** info can viewed, retrieved, updated, or deleted with the function, ammlInfo:

*# look at the metadata associated with the amModelLib*

**ammlInfo**(amml = mymodels)

$owner

[1] "me"

$email

[1] me@somewhere.com

$date.created

[1] "2017-10-04 07:28:28"

*# extract only the owner*

**ammlInfo**(amml = mymodels, x = "owner")

$owner

[1] "me"

*# deletions are done by setting the value associated with a key to NULL*

**ammlInfo**(amml = mymodels) <- **list**(date.created = NULL)

*# update the owner name to Me*, *and add a new metadata element called*

*# organization*

**ammlInfo**(amml = mymodels) <- **list**(owner = "Me", organization = "My Organization")

*# view the result*

**ammlInfo**(amml = mymodels)

$owner

[1] "Me"

$email

[1] me@somewhere.com

$organization

[1] "My Organization"

#### The Data slot

The data slot of an **amModelLib** object contains a list of datasets (analysis inputs), where each dataset is stored as an object of class **amData**. Although the outputs of many analyses will contain the inputs as well (as with the lm and occu examples), the purpose of this new class is to allow the user to store metadata associated with the inputs (datasets) to an analysis.

The key functions associated with **amData** objects include:

- amData—creates an object of class **amData**.
- insertAMModelLib—inserts objects of class **amData** to an **amModelLib** object.
- rmData—deletes an **amData** object from an **amModelLib** object.
- getAMData—extracts ("checks out") an **amData** object from the **amModelLib** object and returns it to R's global environment.

To illustrate, let's add the plant weight data that we analyzed earlier from the lm helpfile. To add the dataset, we first convert the dataset to an **amData** object with the function, amData:

*# Annette Dobson (1990) 'An Introduction to Generalized Linear Models'*.

*# Page 9: Plant Weight Data*.

ctl <- **c**(4.17, 5.58, 5.18, 6.11, 4.5, 4.61, 5.17, 4.53, 5.33, 5.14)

trt <- **c**(4.81, 4.17, 4.41, 3.59, 5.87, 3.83, 6.03, 4.89, 4.32, 4.69)

group <- **gl**(n = 2, k = 10, length = 20, labels = **c**("Ctl", "Trt"))

weight <- **c**(ctl, trt)

*# store the data within a dataframe*

plant.wt <- **data.frame**(weight, group)

*# create an object of class amData*, *which includes the dataset and*

*# corresponding metadata*.

plant.data <- **amData**(data = plant.wt, comment = "Plant dataset from the lm helpfile.", taxa = "plants")

The function amData creates an object of class **amData**, which contains two slots. The first slot is called **data**, which stores the data. In this example, the data are stored as a data frame with 20 observations and 2 variables (the actual plant weight dataset).

The second slot is called **metadata**, which contains a list of metadata associated with this dataset. In the example above, we added metadata fields for comment and taxa. Users should create and standardize their own metadata naming conventions with a key-value syntax. The metadata descriptions (values) can be any length. It is up to the user to determine which metadata to store (the values) and their corresponding names. However, the object creation date, "date.created" is automatically added.

Typing in the name of the **amData** object will display its contents.

*# look at the amData object*

plant.data

An object of class "amData"

Slot "data":

      weight group

1           4.17          Ctl

2           5.58          Ctl

3           5.18          Ctl

4           6.11          Ctl

5           4.50          Ctl

6           4.61          Ctl

7           5.17          Ctl

8           4.53          Ctl

9           5.33          Ctl

10         5.14          Ctl

11         4.81          Trt

12         4.17          Trt

13         4.41          Trt

14         3.59          Trt

15         5.87          Trt

16         3.83          Trt

17         6.03          Trt

18         4.89          Trt

19         4.32          Trt

20         4.69          Trt

Slot "metadata":

$comment

[1] "Plant dataset from the lm helpfile."

$taxa

[1] "plants"

$date

[1] "2017-10-04 07:28:28"

Alternatively, one could see a summary of the object.

*# look at a summary of an amData object*

**summary**(plant.data)

$$\begin{array}{ccc} & \mathrm{weight} & \mathrm{group}\\ \mathrm{Min}.: & 3.590 & \mathrm{Ctl}:10\\ 1\mathrm{st}\mathrm{Qu}.: & 4.388 & \mathrm{Trt}:10\\ \mathrm{Median}: & 4.750 & \\ \mathrm{Mean}: & 4.846\\ 3\mathrm{rd}\mathrm{Qu}.: & 5.218 & \\ \mathrm{Max}.: & 6.110 & \end{array}$$

--- Metadata ---

    name        value

1 comment Plant dataset from the lm helpfile.

2 taxa        plants

3 date        2017-10-04 07:28:28

Here, the data slot in the **amData** object is occupied by a data frame. Thus, the summary function invokes R's summary method for data frames and displays the results. The second portion of the summary output is an abbreviated look at the metadata.

The function insertAMModelLib can be used to insert this object into the **amModelLib**. The **amData** objects to be inserted must be provided as a named list.

*# insert data to amModelLib as a named list*

mymodels <- **insertAMModelLib**(data = **list**(plant.data = plant.data), amml = mymodels)

Typing in the name of the **amModelLib** returns an abbreviated look at the contents:

*# show an abbreviated view of the amModelLib*

mymodels

Description:

[1] This AM Model Library stores analysis inputs (data) and analysis

      outputs (models) associated with the AMModels package vignette.

Info:

    owner

      [1] Me

    email

      [1] me@somewhere.com

    organization

      [1] My Organization

Models:

  --- There are no models ---

Data:

          name          class rows cols package

1 plant.data data.frame        20        2          NA

Under the "Data:" section, we see the name of each **amData** object, its class, and the package of origin (if it is readily available). Rows and columns are also provided for objects that contain data frames.

The metadata associated with all **amData** objects or a specific **amData** objects can be viewed or set with the dataMeta function.

*# view all metadata associated with datasets*

**dataMeta**(amml = mymodels)

$plant.data

$plant.data$comment

[1] "Plant dataset from the lm helpfile."

$plant.data$taxa

[1] "plants"

$plant.data$date

[1] "2017-10-04 07:28:28"

*# view metadata associated with the amData object*, *plant.data*

**dataMeta**(amml = mymodels, x = "plant.data")

$comment

[1] "Plant dataset from the lm helpfile."

$taxa

[1] "plants"

$date

[1] "2017-10-04 07:28:28"

*# add some additional metadata to an amData object in the form of a named*

*# list*

**dataMeta**(amml = mymodels, x = "plant.data") <- **list** (url = "https://stat.ethz.ch/R-manual/R-devel/library/stats/html/lm.html",

group = "The column named 'group' identifies which group a sample belongs to, where Ctl = control group and Trt = treatment group.",

weight = "The column named 'weight' provides total biomass of the sample, in grams.")

The dataMeta function can also be used to quickly copy existing metadata to a new **amData** object. For instance, agencies or organizations that produce the same analyses year after year can create a new **amData** object that includes an updated dataset, but utilizes the metadata from a previous year's **amData** object.

*# recall our original plant data is from the lm helpfile*

plant.wt <- **data.frame**(group, weight)

*# add a few new records of plant data*

new.plant.data <- **data.frame**(group = **c**("Ctl", "Trt"), weight = **c**(4.5, 3.83))

*# combine data to make an updated plant dataset*

updated.plant.data <- **rbind**(plant.wt, new.plant.data)

*# extract the metadata from the existing amData object*, *plant*.*data*

metadata <- **dataMeta**(amml = mymodels, x = "plant.data")

*# create new amData object that combines the new data with the existing metadata*

updated.plant.data <- **amData**(data = updated.plant.data, metadata)

*# insert the amData object to the AM model library as a named list*

mymodels <- **insertAMModelLib**(data = **list**(updated.plant.data = updated.plant.data), amml = mymodels)

*# view metadata associated with the new amData object*, *updated*.*plant*.*data*

**dataMeta**(amml = mymodels, x = "updated.plant.data")

$comment

[1] "Plant dataset from the lm helpfile."

$taxa

[1] "plants"

$date

[1] "2017-10-04 07:28:28"

$url

[1] https://stat.ethz.ch/R-manual/R-devel/library/stats/html/lm.html

$group

[1] "The column named 'group' identifies which group a sample belongs to, where Ctl = control group and Trt = treatment group."

$weight

[1] "The column named 'weight' provides total biomass of the sample, in grams."

Multiple **amData** objects can be inserted to an **amModelLib** simultaneously using the same process. For example, below we create a data frame containing our apple infection data (called apples), and a second data frame (called sim.covs) that is simulated with the function, simCovariate.

*# create apple infestation data frame*

apples <- **data.frame**(n = 100, y = 25)

*# create a list of uncorrelated covariates*

cov.list <- **list**(unif1 = **list**(dist = "runif", min = 0, max = 10, seed = 334,

round = 0), unif2 = **list**(dist = "runif", min = 0, max = 10, seed = 668,

round = 0), norm1 = **list**(dist = "normal", mean = 10, sd = 2, seed = 10,

round = 1), norm2 = **list**(dist = "normal", mean = 50, sd = 10, seed = 15,

round = 2), beta1 = **list**(dist = rbeta, shape1 = 2, shape2 = 1, seed = 1002),

binom1 = **list**(dist = "bin", size = 20, prob = 0.5, seed = 561), bern1 = **list**(dist = "bernoulli", size = 1, prob = 0.5, seed = 6))

*# generate a data frame with the covariate list and show*

(sim.covs <- **simCovariate**(cov.list = cov.list, n = 10, add.yr = TRUE))

$$\begin{array}{ccccccccc} & \mathrm{unif}1 & \mathrm{unif}2 & \mathrm{norm}1 & \mathrm{norm}2 & \mathrm{beta}1 & \mathrm{binom}1 & \mathrm{bern}1 & \mathrm{yr}\\ 1 & 7 & 6 & 9.1 & 44.07 & 0.9008661 & 8 & 1 & 1\\ 2 & 8 & 7 & 9.0 & 31.52 & 0.6645892 & 11 & 1 & 2\\ 3 & 3 & 4 & 11.4 & 39.52 & 0.9618653 & 13 & 1 & 3\\ 4 & 1 & 8 & 6.5 & 45.96 & 0.8872998 & 8 & 1 & 4\\ 5 & 10 & 4 & 9.4 & 44.17 & 0.5601055 & 11 & 0 & 5\\ 6 & 8 & 7 & 7.6 & 43.26 & 0.9934818 & 9 & 1 & 6\\ 7 & 6 & 8 & 10.9 & 42.66 & 0.6639714 & 11 & 1 & 7\\ 8 & 0 & 1 & 11.2 & 53.89 & 0.6274681 & 6 & 0 & 8\\ 9 & 2 & 3 & 8.5 & 51.77 & 0.6857797 & 11 & 0 & 9\\ 10 & 3 & 1 & 9.5 & 63.01 & 0.7370935 & 11 & 1 & 10 \end{array}$$

To add these two datasets to our **amModelLib**, we first convert each to an object of class **amData** with the function, **amData**, and then insert it to the **amModelLib** with the insertAMModelLib function.

*# create an amData object containing the apple data*.

apple.data <- **amData**(data = apples, comment = "Apple infestation dataset for Bayesian analysis.")

*# create an amData object containing the simulated covariate data*.

sim.data <- **amData**(data = sim.covs, comment = "Simulated covariate dataset.")

*# add the two amData objects to the amModelLib; remember it must be named list*.

mymodels <- **insertAMModelLib**(data = **list**(apple.data = apple.data, sim.data = sim.data), amml = mymodels)

*# show the contents; note the library now contains 4 datasets*.

mymodels

Description:

[1] This AM Model Library stores analysis inputs (data) and analysis

      outputs (models) associated with the AMModels package vignette.

Info:

    owner

      [1] Me

    email

      [1] me@somewhere.com

    organization

      [1] My Organization

Models:

  --- There are no models ---

Data:

$$\begin{array}{cccccc} & \;\mathrm{name} & \;\mathrm{class} & \;\mathrm{rows} & \mathrm{cols} & \mathrm{package}\\ 1 & \;\mathrm{plant}.\mathrm{data} & \mathrm{data}.\mathrm{frame} & 20 & 2 & \mathrm{NA}\\ 2 & \mathrm{updated}.\mathrm{plant}.\mathrm{data} & \mathrm{data}.\mathrm{frame} & 22 & 2 & \mathrm{NA}\\ 3 & \;\mathrm{apple}.\mathrm{data} & \mathrm{data}.\mathrm{frame} & 1 & 2 & \mathrm{NA}\\ 4 & \;\mathrm{sim}.\mathrm{data} & \mathrm{data}.\mathrm{frame} & 10 & 8 & \mathrm{NA} \end{array}$$

Datasets are not limited to data frames. To demonstrate, we now add the chorus frog unmarked frame object to an **amData** object and add it to our **amModelLib**.

*# load the chorus frog data*

**data**(frogs)

*# create an unmarked frame*

pferUMF <- **unmarkedFrameOccu**(pfer.bin)

*# get the number of sites in which frogs were surveyed*

num.sites <- **numSites**(pferUMF)

*# add a fake site level covariate called sitevar1 for illustration*

*# specify a seed for a random number generator; used for reproducibility*.

**set.seed**(20)

site.sums <- **rowSums**(pferUMF@y, na.rm = T)

sitevar1 = **round**(**rnorm**(n = num.sites, mean = **ifelse**(test = site.sums > 0, yes = 15, no = 10), sd = 3), 2)

**siteCovs**(pferUMF) <- **data.frame**(sitevar1)

*# create an object of class amData*

frog.data <- **amData**(data = pferUMF, comment = "Chorus frog dataset from the package unmarked.", taxa = "Chorus Frog", url = "http://www.rdocumentation.org/packages/unmarked/versions/0.11-0/topics/occu")

*# the summary function would show the results of the summary method in package unmarked summary(frog*.*data)*

*# add the amData to the amModelLib as a named list*

mymodels <- **insertAMModelLib**(data = **list**(frog.data = frog.data), amml = mymodels)

Our **amModelLib** now consists of five **amData** objects within the data slot:

*# show the contents of an amModelLib object*

mymodels

Description:

[1] This AM Model Library stores analysis inputs (data) and analysis

      outputs (models) associated with the AMModels package vignette.

Info:

    owner

      [1] Me

    email

      [1] me@somewhere.com

    organization

      [1] My Organization

Models:

  --- There are no models ---

Data:

$$\begin{array}{cccccc} & \;\mathrm{name} & \;\mathrm{class} & \;\mathrm{rows} & \mathrm{cols} & \mathrm{package}\\ 1 & \;\mathrm{plant}.\mathrm{data} & \;\mathrm{data}.\mathrm{frame} & 20 & 2 & <\mathrm{NA}>\\ 2 & \mathrm{updated}.\mathrm{plant}.\mathrm{data} & \;\mathrm{data}.\mathrm{frame} & 22 & 2 & <\mathrm{NA}>\\ 3 & \;\mathrm{apple}.\mathrm{data} & \;\mathrm{data}.\mathrm{frame} & 1 & 2 & <\mathrm{NA}>\\ 4 & \;\mathrm{sim}.\mathrm{data} & \;\mathrm{data}.\mathrm{frame} & 10 & 8 & <\mathrm{NA}>\\ 5 & \;\mathrm{frog}.\mathrm{data} & \mathrm{unmarkedFrameOccu} & \mathrm{NA} & \mathrm{NA} & \mathrm{unmarked} \end{array}$$

Use the lsData function to retrieve the names of the **amData** objects in an **amModelLib**, where **ls** indicates list (in the general sense):

*# retrieve the names of the amData objects*

**lsData**(mymodels)

[1] "plant.data"            "updated.plant.data" "apple.data"

[4] "sim.data"                "frog.data"

For objects of class **amModelLib**, the summary function will provide a summary of the contents of the **amModelLib** as shown above, but will also display an abbreviated view of the metadata.

*# run the summary method an amModelLib object*

**summary**(mymodels)

Description:

[1] This AM Model Library stores analysis inputs (data) and analysis

      outputs (models) associated with the AMModels package vignette.

Info:

    owner

      [1] Me

    email

      [1] me@somewhere.com

    organization

      [1] My Organization

  --- There are no models ---

--- Data Names and Indices ---

$$\begin{array}{cccccc} & \;\mathrm{name} & \;\mathrm{class} & \;\mathrm{rows} & \mathrm{cols} & \mathrm{package}\\ 1 & \;\mathrm{plant}.\mathrm{data} & \;\mathrm{data}.\mathrm{frame} & 20 & 2 & <\mathrm{NA}>\\ 2 & \mathrm{updated}.\mathrm{plant}.\mathrm{data} & \;\mathrm{data}.\mathrm{frame} & 22 & 2 & <\mathrm{NA}>\\ 3 & \;\mathrm{apple}.\mathrm{data} & \;\mathrm{data}.\mathrm{frame} & 1 & 2 & <\mathrm{NA}>\\ 4 & \;\mathrm{sim}.\mathrm{data} & \;\mathrm{data}.\mathrm{frame} & 10 & 8 & <\mathrm{NA}>\\ 5 & \;\mathrm{frog}.\mathrm{data} & \mathrm{unmarkedFrameOccu} & \mathrm{NA} & \mathrm{NA} & \mathrm{unmarked} \end{array}$$

--- Data metadata ---

[[1]] plant.data

namevalue1commentPlantdatasetfromthelmhelpfile2taxaplants3date2017-10-0407:28:284urlhttps://stat.ethz.ch/R-manual/R-devel/library/stats/html/lm…5groupThecolumnnamed'group'identifieswhichgroupasamplebel…6weightThecolumnnamed'weight'providestotalbiomassofthesamp…

[[2]] updated.plant.data

namevalue1commentPlantdatasetfromthelmhelpfile2taxaplants3date2017-10-0407:28:284urlhttps://stat.ethz.ch/R-manual/R-devel/library/stats/html/lm…5groupThecolumnnamed'group'identifieswhichgroupasamplebel…6weightThecolumnnamed'weight'providestotalbiomassofthesamp…

[[3]] apple.data

$$\begin{array}{ccc} & \mathrm{name}\; & \mathrm{value}\;\\ 1 & \mathrm{comment} & \mathrm{Apple}\;\mathrm{infestation}\;\mathrm{dataset}\;\mathrm{for}\;\mathrm{Bayesian}\;\mathrm{analysis}.\\ 2 & \mathrm{date}\; & 2017-10-04\;07:28:28\; \end{array}$$

[[4]] sim.data

$$\begin{array}{ccc} & \mathrm{name}\; & \mathrm{value}\;\\ 1 & \mathrm{comment} & \mathrm{Simulated}\;\mathrm{covariate}\;\mathrm{dataset}.\\ 2 & \mathrm{date}\; & 2017-10-04\;07:28:28\; \end{array}$$

[[5]] frog.data

$$\begin{array}{ccc} & \mathrm{name}\; & \mathrm{value}\;\\ 1 & \mathrm{comment} & \mathrm{Chorus}\;\mathrm{frog}\;\mathrm{dataset}\;\mathrm{from}\;\mathrm{the}\;\mathrm{package}\;\mathrm{unmarked}.\;\\ 2 & \mathrm{taxa}\; & \mathrm{Chorus}\;\mathrm{Frog}\;\\ 3 & \mathrm{url}\; & \;\mathrm{http}://\mathrm{www}.\mathrm{rdocumentation}.\mathrm{org}/\mathrm{packages}/\mathrm{unmarked}/\mathrm{versions}/0.1\\ 4 & \mathrm{date}\; & 2017-10-04\;07:28:29\; \end{array}$$

The summary output is intended to let the user scan the names and indices of the models and datasets within the **amModelLib** object, and then use the index to quickly find the metadata associated with each object. Notice that the summary function truncates the metadata values.

To retrieve a specific **amData** object and return the object to R's global environment in its original class, the getAMData accessor function can be used. Let's extract the plant data with the getAMData function, which requires the name of the **amData** object. The argument, **as.list** indicates how the object is to be returned: FALSE will return the data in its original class, while TRUE will return a list containing the data in its original class and the metadata.

*# retrieve the frog*.*data to R's global environment (this does not delete it from the library)*

extracted.plant.data <- **getAMData**(x = "plant.data", amml = mymodels, as.list = FALSE)

*# the extracted data are returned in their original form*

**class**(extracted.plant.data)

[1] "data.frame"

As a second example, we will extract the chorus frog data with the getAMData function as a list:

*# retrieve the frog*.*data to R's global environment as a list containing data and metadata*

extracted.frog.data <- **getAMData**(x = "frog.data", amml = mymodels, as.list = TRUE)

*# view the first three records of the extracted*.*frog*.*data*

**lapply**(X = extracted.frog.data, FUN = head, n = 3)

$data

Data frame representation of unmarkedFrame object.

$$\begin{array}{cccccccc} & y.1 & y.2 & y.3 & \mathrm{sitevar}1 & \mathrm{obsvar}1.1 & \mathrm{obsvar}1.2 & \mathrm{obsvar}1.3\\ 1 & 1 & 0 & \mathrm{NA} & 13.02 & 1 & 2 & 3\\ 2 & 1 & 0 & 0 & 12.92 & 1 & 2 & 3\\ 3 & 1 & 0 & 0 & 17.75 & 1 & 2 & 3 \end{array}$$

$metadata

$metadata$comment

[1] "Chorus frog dataset from the package unmarked."

$metadata$taxa

[1] "Chorus Frog"

$metadata$url

[1] "http://www.rdocumentation.org/packages/unmarked/versions/0.11-0/topics/occu"

Notice the returned object is a list containing two elements. The first list element is named data, which is of class of **unmarkedFrameOccu** generated by the package, unmarked. The second list element is another list providing the metadata associated with the dataset.

To remove a specific **amData** object from the **amModelLib**, use the rmData function. **amData** objects can be deleted by index or by name. Here, we delete the 'sim.data' **amData** object by referencing its current index (4).

*# remove the amData object*, *'sim*.*data' by index*

mymodels <- **rmData**(amml = mymodels, x = 4)

*# notice the sim*.*data amModel object has been removed*, *leaving 4 datasets*

**lsData**(mymodels)

[1] "plant.data"            "updated.plant.data" "apple.data"

[4] "frog.data"

Note that all indices have shifted as a result. We can also delete by name. Here, we delete the **amData** 'updated.plant.data':

*# remove the amModel 'updated.plant.data' from the amModelLib*

mymodels <- **rmData**(amml = mymodels, x = "updated.plant.data")

*# notice the updated*.*plant*.*data amModel object has been removed*, *leaving 3 datasets*

**lsData**(mymodels)

[1] "plant.data" "apple.data" "frog.data"

#### The Models slot

Models (analysis outputs) are stored in the model slot of the **amModelLib** object, and the model slot is a list of objects of class **amModel**. As with the **amData** class detailed above, the purpose of this class is to store not only the analysis outputs, but also metadata about the outputs. The key functions associated with **amModel** objects (Supplement 1) include:

- amModel—creates an object of class **amModel**.
- insertAMModelLib—inserts objects of class **amModel** to a **amModelLib** object.
- rmModel—deletes an **amModel** object.
- getAMModel—extracts an **amModel** object from the **amModelLib** object and returns it to the global environment in its original form.

To illustrate, first we will retrieve our plant data from the library, and then analyze plant weights with the function, lm and store the results in an object of class **lm** named **lm.D9**. Next, we will use the amModel function to convert the analysis outputs to an **amModel** object (including metadata), and will then add this model to our **amModelLib** with the insertAMModelLib function. As with datasets, models must be supplied as a named list and a user can enter any metadata using a key-value syntax.

*# retrieve the plant data from the library*

plant.data <- **getAMData**(amml = mymodels, x = "plant.data", as.list = FALSE)

*# run the analysis*

lm.D9 <- **lm**(weight ~ group, data = plant.data)

*# create an amModel and add metadata*

plant.model <- **amModel**(model = lm.D9, comment = "Analysis from lm helpfile",

data = "plant.data")

*# insert the model to the amModelLib as a named list*

mymodels <- **insertAMModelLib**(models = **list**(plant.model = plant.model), amml = mymodels)

You may recall that the **lm.D9** object contains a rich amount of information about the linear model analysis, including the data inputs. The example above adds **lm.D9** to an **amModel** object, and supplies additional metadata about the analysis itself.

In this example, the metadata contains an (optional) element named **data**, which points to the name of the **amData** dataset that was used to create that model. **The relationship established by this pairing is completely informal and no checking is performed to verify the existence or compatibility of the data.** However, accessor functions will look for the keyword "data" in the model's metadata, and will retrieve the dataset that is linked to the model if such a pairing is indicated. The "data" metadata element is currently the only element that will trigger a relationship pairing. [Future versions of AMModels will include a "prior" metadata element that pairs posterior models with their prior models.]

Typing in the name of the **amModel** object will show the object according to the object's original class. Alternately, a user can invoke the summary method to look at the **amModel** object, which returns a summary of the model and its associated metadata.

*# look at the summarized snapshot of an amModel object*

**summary**(plant.model)

Call:

lm(formula = weight ~ group, data = plant.data)

Residuals:

$$\begin{array}{ccccc} \;\mathrm{Min} & \;1Q & \mathrm{Median} & \;3Q & \;\mathrm{Max}\\ -1.0710 & -0.4938 & 0.0685 & 0.2462 & 1.3690 \end{array}$$

Coefficients:

$$\begin{array}{cccccc} & \mathrm{Estimate}\mathrm{Std}. & \mathrm{Error} & t\;\mathrm{value} & \;\mathrm{Pr}(>|t|) & \\ \left(\mathrm{Intercept}\right) & 5.0320 & 0.2202 & 22.850 & 9.55e-15 & ***\\ \mathrm{groupTrt}\; & -0.3710 & 0.3114 & -1.191 & \;0.249 & \end{array}$$

Signif. codes:      0 '***' 0.001 '**' 0.01 '*' 0.05 '.' 0.1 ' ' 1

Residual standard error: 0.6964 on 18 degrees of freedom

Multiple R-squared: 0.07308, Adjusted R-squared: 0.02158

F-statistic: 1.419 on 1 and 18 DF, p-value: 0.249

--- Metadata ---

$$\begin{array}{ccc} & \mathrm{name}\; & \mathrm{value}\;\\ 1 & \mathrm{comment} & \mathrm{Analysis}\;\mathrm{from}\;\mathrm{lm}\;\mathrm{helpfile}\\ 2 & \mathrm{data}\; & \mathrm{plant}.\mathrm{data}\;\\ 3 & \mathrm{date}\; & 2017-10-04\;07:28:29\; \end{array}$$

Here, because the model slot in the **amModel** object is occupied by an object of class **lm**, the call is passed to the summary method defined for **lm** objects and the results are displayed.

The same steps are used to add the chorus frog occupancy model to the **amModelLib**. Here, we add the results of our two occupancy models (**fm1** and **fm2**) by first creating **amModel** objects with metadata, and then inserting them to the **amModelLib** with the insertAMModelLib function.

*# retrieve the frog data from the library (an unmarkedFrame object)*

frog.data <- **getAMData**(amml = mymodels, x = "frog.data", as.list = FALSE)

*# run the first analysis and store the results as fm1*

fm1 <- **occu**(formula = ~obsvar1 ~ sitevar1, data = frog.data)

*# create an amModel object for the first analysis and add metadata*

frog.model1 <- **amModel**(model = fm1, comment = "Occupancy as a function of sitevar1 and detection as a function of obsvar1.", data = "frog.data")

*# run the second analysis (the intercept only model) and store the results as fm2*

fm2 <- **occu**(formula = ~1 ~ 1, data = frog.data)

*# create an amModel object for the second analysis and add metadata*

frog.model2 <- **amModel**(model = fm2, comment = "Occupancy as a function of no covariates and detection as a function of no covariates", data = "frog.data")

*# insert both models to the amModelLib as a named list*

mymodels <- **insertAMModelLib**(models = **list**(frog.model1 = frog.model1, frog.model2 = frog.model2), amml = mymodels)

*# show the contents of the amModelLib object*

mymodels

Description:

[1] This AM Model Library stores analysis inputs (data) and analysis

      outputs (models) associated with the AMModels package vignette.

Info:

    owner

      [1] Me

    email

      [1] me@somewhere.com

    organization

      [1] My Organization

Models:

$$\begin{array}{cccc} & \;\mathrm{name} & \;\mathrm{class} & \mathrm{package}\\ 1 & \mathrm{plant}.\mathrm{model} & \;\mathrm{lm} & <\mathrm{NA}>\\ 2 & \mathrm{frog}.\mathrm{model}1 & \mathrm{unmarkedFitoccu} & \mathrm{unmarked}\\ 3 & \mathrm{frog}.\mathrm{model}2 & \mathrm{unmarkedFitoccu} & \mathrm{unmarked} \end{array}$$

Data:

$$\begin{array}{cccccc} & \;\mathrm{name} & \;\mathrm{class} & \mathrm{rows} & \mathrm{cols} & \mathrm{package}\\ 1 & \mathrm{plant}.\mathrm{data} & \;\mathrm{data}.\mathrm{frame} & 20 & 2 & \;<\mathrm{NA}>\\ 2 & \mathrm{apple}.\mathrm{data} & \;\mathrm{data}.\mathrm{frame} & 1 & 2 & \;<\mathrm{NA}>\\ 3 & \mathrm{frog}.\mathrm{data} & \mathrm{unmarkedFrameOccu} & \mathrm{NA} & \mathrm{NA} & \mathrm{unmarked} \end{array}$$

Finally, let's add two Bayesian apple infestation rate models. Recall that this analysis is a user-defined model that has not been formally conducted with a dedicated R package, which would be a normal course of action. Here, we will link the second model to the first model using the 'prior' metadata keyword, and link the second model's data to the 'apple.data' **amData** object using the 'data' metadata keyword.

*# create apple*.*m1 as a list*

apple.m1 <- **list**(distribution = "beta", parameters = **list**(alpha = 2, beta = 3))

*# create an amModel object for apple*.*m1*

apple.m1 <- **amModel**(model = apple.m1, comment = "Bayes model 1 for apple infestation")

*# create apple*.*m2 as a list*

apple.m2 <- **list**(distribution = "beta", posteriors = **list**(alpha = 27, beta = 78))

*# create an amModel object for apple*.*m2; link this model to apple*.*m1 and also associate the dataset*.

apple.m2 <- **amModel**(model = apple.m2, comment = "Bayes model 2 for apple infestation", prior = "apple.m1", data = "apple.data")

*# insert both models to the amModelLib object as a named list*

mymodels <- **insertAMModelLib**(models = **list**(apple.m1 = apple.m1, apple.m2 = apple.m2), amml = mymodels)

Our **amModelLib** object now contains five **amModel** objects and three **amData** objects.

*# show the contents of the amModelLib object*

mymodels

Description:

[1] This AM Model Library stores analysis inputs (data) and analysis

      outputs (models) associated with the AMModels package vignette.

Info:

    owner

      [1] Me

    email

      [1] me@somewhere.com

    organization

      [1] My Organization

Models:

$$\begin{array}{cccc} & \;\mathrm{name} & \;\mathrm{class} & \mathrm{package}\\ 1 & \mathrm{plant}.\mathrm{model} & \;\mathrm{lm} & \;<\mathrm{NA}>\\ 2 & \mathrm{frog}.\mathrm{model}1 & \mathrm{unmarkedFitOccu} & \mathrm{unmarked}\\ 3 & \mathrm{frog}.\mathrm{model}2 & \mathrm{unmarkedFitOccu} & \mathrm{unmarked}\\ 4 & \mathrm{apple}.m1 & \;\mathrm{list} & \;<\mathrm{NA}>\\ 5 & \mathrm{apple}.m2 & \;\mathrm{list} & \;<\mathrm{NA}> \end{array}$$

Data:

$$\begin{array}{cccccc} & \;\mathrm{name} & \;\mathrm{class} & \mathrm{rows} & \mathrm{cols} & \;\mathrm{package}\\ 1 & \mathrm{plant}.\mathrm{data} & \;\mathrm{data}.\mathrm{frame} & 20 & 2 & \;<\mathrm{NA}>\\ 2 & \mathrm{apple}.\mathrm{data} & \;\mathrm{data}.\mathrm{frame} & 1 & 2 & \;<\mathrm{NA}>\\ 3 & \mathrm{frog}.\mathrm{data} & \mathrm{unmarkedFrameOccu} & \mathrm{NA} & \mathrm{NA} & \mathrm{unmarked} \end{array}$$

The summary function would display the same information, but additionally provide the metadata associated with each **amModel** and **amData** object.

Use the lsModels function to retrieve the names of the **amModel** objects in an **amModelLib**:

*# list the amModel objects within the library*

**lsModels**(mymodels)

[1] "plant.model" "frog.model1" "frog.model2" "apple.m1"        "apple.m2"

The modelMeta function is analogous to the dataMeta function and allows metadata to be viewed or set after the model has been inserted into the **amModelLib** object. Although we do not provide an example, it is possible to store the R code used to generate the model as metadata to enhance analytic reproducibility.

To retrieve ("check out") a specific **amModel** object and return its original class to R's global environment, the getAMModel accessor function can be used. Let's extract the first frog model with the getAMModel function, which requires the name of the **amModel** object. The optional argument, **as.list** indicates how the object is to be returned: FALSE (default) will return the model in its original class, while TRUE will return a list containing the model in its original class and the metadata.

*# retrieve frog*.*model1; this does not remove the model from the library*

extracted.frog.model1 <- **getAMModel**("frog.model1", amml = mymodels, as.list = FALSE)

*# the extracted model is returned in its original form*

**class**(extracted.frog.model1)

[1] "unmarkedFitOccu"

attr(,"package")

[1] "unmarked"

To remove a specific **amModel** object from the **amModelLib**, use rmModel function. As with rmData, a model can be deleted by index or by name. Here, we delete the **amModel** 'apple.m2':

*# remove the amModel 'apple*.*m2' from the amModelLib*

mymodels <- **rmModel**(mymodels, "apple.m2")

*# show the amModelLib*

mymodels

Description:

[1] This AM Model Library stores analysis inputs (data) and analysis

      outputs (models) associated with the AMModels package vignette.

Info:

    owner

      [1] Me

    email

      [1] me@somewhere.com

    organization

      [1] My Organization

Models:

$$\begin{array}{cccc} & \;\mathrm{name} & \;\mathrm{class} & \mathrm{package}\\ 1 & \mathrm{plant}.\mathrm{model} & \;\mathrm{lm} & \;<\mathrm{NA}>\\ 2 & \mathrm{frog}.\mathrm{model}1 & \mathrm{unmarkedFitOccu} & \mathrm{unmarked}\\ 3 & \mathrm{frog}.\mathrm{model}2 & \mathrm{unmarkedFitOccu} & \mathrm{unmarked}\\ 4 & \mathrm{apple}.m1 & \;\mathrm{list} & \;<\mathrm{NA}> \end{array}$$

Data:

$$\begin{array}{cccccc} & \;\mathrm{name} & \;\mathrm{class} & \mathrm{rows} & \mathrm{cols} & \;\mathrm{package}\\ 1 & \mathrm{plant}.\mathrm{data} & \;\mathrm{data}.\mathrm{frame} & 20 & 2 & \;<\mathrm{NA}>\\ 2 & \mathrm{apple}.\mathrm{data} & \;\mathrm{data}.\mathrm{frame} & 1 & 2 & \;<\mathrm{NA}>\\ 3 & \mathrm{frog}.\mathrm{data} & \mathrm{unmarkedFrameOccu} & \mathrm{NA} & \mathrm{NA} & \mathrm{unmarked} \end{array}$$

Note that all indices have shifted as a result. The shifting indices illustrate a critical concept: *never* link models and data by index number as indices will shift as items are inserted and removed from the **amModelLib** object. Users who link models with data or models with models should do so by name, and the names should be treated as permanent names to retain the integrity of the library.

### Searching and subsetting the amModelLib

Users may create as many **amModelLibs** as needed to help organize a variety of models. As the number of models and datasets grows within an **amModelLib** object, it may be useful to search for objects within it and subset the **amModelLib** object if necessary. The function, grepAMModelLib can be used for this purpose; it returns an object of class **amModelLib** containing the matches to the search (if any).

The function uses grep to recursively search for a pattern in a given **amModelLib** object; the pattern argument may therefore be a simple string or a regular expression. The user may search both the model and data slots ('all'), or either the model or the data slot by specifying a **search** argument; omitting the **search** argument will default to 'all'.

**args**(grepAMModelLib)

function (pattern, amml, search = c("all", "model", "data"),…)

NULL

For example, here we search through all **amData** objects that include the term **frog**.

*# search for data containing the word 'frog'*

**grepAMModelLib**(pattern = "frog", amml = mymodels, search = "data")

Description:

[1] This AM Model Library stores analysis inputs (data) and analysis

      outputs (models) associated with the AMModels package vignette.

Info:

    owner

      [1] Me

    email

      [1] me@somewhere.com

    organization

      [1] My Organization

Models:

$$\begin{array}{cccc} & \;\mathrm{name} & \;\mathrm{class} & \;\mathrm{package}\\ 1 & \mathrm{frog}.\mathrm{model}1 & \mathrm{unmarkedFitOccu} & \mathrm{unmarked}\\ 2 & \mathrm{frog}.\mathrm{model}2 & \mathrm{unmarkedFitOccu} & \mathrm{unmarked} \end{array}$$

Data:

$$\begin{array}{cccccc} & \;\mathrm{name} & \;\mathrm{class} & \mathrm{rows} & \mathrm{cols} & \;\mathrm{package}\\ 1 & \mathrm{frog}.\mathrm{data} & \mathrm{unmarkedFrameOccu} & \;\mathrm{NA} & \mathrm{NA} & \mathrm{unmarked} \end{array}$$

The results show an (unsaved) **amModelLib** object which contains an **amData** object called 'frog.data'. Because the frog dataset was linked to frog.model1 and frog.model2 with the "data" metadata element, the **amModelLib** object also contains two **amModel** objects.

Alternatively, the **amModelLib** can be subset with list subsetting. List subsetting methods for **amModelLib** objects also attempt to keep the data with the models if they have been paired by matching against the 'data' metadata element. Here, we subset the library by the name, "plant.model", store the result as 'mymodels2', and verify that the library contains both the model and the linked dataset.

*# create new amModelLib named plantAnalyses that includes only the plant models*

plantAnalyses <- mymodels[**c**("plant.model")]

*# update the description*

**ammlDesc**(amml = plantAnalyses) <- "This AM Model Library stores analysis inputs (data) and analysis     outputs (models) associated with the plant data in the lm helpfile."

*# show the new library*

plantAnalyses

Description:

[1] This AM Model Library stores analysis inputs (data) and analysis

      outputs (models) associated with the plant data in the lm

      helpfile.

Info:

    owner

      [1] Me

    email

      [1] me@somewhere.com

    organization

      [1] My Organization

Models:

$$\begin{array}{cccc} & \;\mathrm{name} & \mathrm{class} & \mathrm{package}\\ 1 & \mathrm{plant}.\mathrm{model} & \;\mathrm{lm} & \mathrm{NA} \end{array}$$

Data:

$$\begin{array}{cccccc} & \;\mathrm{name} & \;\mathrm{class} & \mathrm{rows} & \mathrm{cols} & \;\mathrm{package}\\ 1 & \mathrm{plant}.\mathrm{data} & \mathrm{data}.\mathrm{frame} & \;20 & \;2 & \;\mathrm{NA} \end{array}$$

The grepAMModelLib function is intended to subset the **amModelLib** object to allow users to locate datasets and associated models, and vice versa. However, if the subset library is stored, such as in the example above, some models and datasets will be contained in multiple libraries, which could lead to confusion (e.g., the plant analysis is now stored in two libraries, **plantAnalyses** and **mymodels**).

To avoid such duplication, the grepAMModelLib function can be used to split an existing **amModelLib**. For example, suppose we decide that the frog analyses should be stored in a separate library. The following code will search for the frog models and datasets, create a new library called "chorusFrogLibrary", and remove them from the original library:

*# pull out models and data containing the word 'frog' from the library*, *mymodels*

chorusFrogLibrary <- **grepAMModelLib**(pattern = "frog", amml = mymodels, search = "all")

*# update the library description*

**ammlDesc**(amml = chorusFrogLibrary) <- "This AM Model Library stores analysis inputs (data) and analysis outputs (models) associated with chorus frog research and management."

*# remove the amModels now in frog*.*library from mymodels*

mymodels <- **rmModel**(amml = mymodels, x = **lsModels**(chorusFrogLibrary))

*# remove the amData now in frog*.*library from mymodels*

mymodels <- **rmData**(amml = mymodels, x = **lsData**(chorusFrogLibrary))

*# look at the frog*.*library*

chorusFrogLibrary

Description:

[1] This AM Model Library stores analysis inputs (data) and analysis

      outputs (models) associated with chorus frog research and

      management.

Info:

    owner

      [1] Me

    email

      [1] me@somewhere.com

    organization

      [1] My Organization

Models:

$$\begin{array}{cccc} & \;\mathrm{name} & \;\mathrm{class} & \;\mathrm{package}\\ 1 & \mathrm{frog}.\mathrm{model}1 & \mathrm{unmarkedFitOccu} & \mathrm{unmarked}\\ 2 & \mathrm{frog}.\mathrm{model}2 & \mathrm{unmarkedFitOccu} & \mathrm{unmarked} \end{array}$$

Data:

$$\begin{array}{cccccc} & \;\mathrm{name} & \;\mathrm{class} & \mathrm{rows} & \mathrm{cols} & \;\mathrm{package}\\ 1 & \mathrm{frog}.\mathrm{data} & \mathrm{unmarkedFrameOccu} & \;\mathrm{NA} & \mathrm{NA} & \mathrm{unmarked} \end{array}$$

*# look at the original mymodels library; notice the frog analyses have been removed*, *while the plant analyses have been retained because they were not removed when subsetting the plant library*

mymodels

Description:

[1] This AM Model Library stores analysis inputs (data) and analysis

      outputs (models) associated with the AMModels package vignette.

Info:

    owner

      [1] Me

    email

      [1] me@somewhere.com

    organization

      [1] My Organization

Models:

$$\begin{array}{cccc} & \;\mathrm{name} & \mathrm{class} & \mathrm{package}\\ 1 & \mathrm{plant}.\mathrm{model} & \;\mathrm{lm} & \;\mathrm{NA}\\ 2 & \;\mathrm{apple}.m1 & \;\mathrm{list} & \;\mathrm{NA} \end{array}$$

Data:

$$\begin{array}{cccccc} & \;\mathrm{name} & \;\mathrm{class} & \mathrm{rows} & \mathrm{cols} & \mathrm{package}\\ 1 & \mathrm{plant}.\mathrm{data} & \mathrm{data}.\mathrm{frame} & \;20 & 2 & \;\mathrm{NA}\\ 2 & \mathrm{apple}.\mathrm{data} & \mathrm{data}.\mathrm{frame} & \;1 & 2 & \;\mathrm{NA} \end{array}$$

### Saving the amModelLib object

You may save your **amModelLib** with either the save or saveRDS functions from the base package.

*# save mymodels to an.rda file (which can store multiple objects or multiplelibraries)*

**save**(mymodels, file = "mymodels.rda")

*# save frog*.*analyses to an*.*RDS file (which can store a single library)*

**saveRDS**(chorusFrogLibrary, file = "chorusFrogLibrary.RDS")

The file argument should include the filepath where the .RData or.rda file will live. To call up the **amModelLib**, the load or readRDS function can be used.

*# load a stored*.*rda file*

**load**("mymodels.rda")

*# load a stored*.*rds file (which will store a single object*, *in this case a single ammodelLib)*

chorusFrogLibrary <- **readRDS**("chorusFrogLibrary.RDS")

We envision that saved **amModelLib** objects will be used repeatedly as they contain a storehouse of models and datasets that can be retrieved for future use.

### amModelLib organization

Users may maintain multiple **amModelLib**s to best meets their needs. For example, a single **amModelLib** may be used to:

- store models associated with a specific set of analyses, such as those included in an annual report, a scientific paper, or a monitoring program;
- store models associated with a specific parameter of interest (e.g., all models related to apple infestation rate);
- store models associated with a specific species or research project (e.g., the chorus frog analyses);
- store models by model type (all occupancy models);
- store all models by a particular person;
- etc.

Trial and error may reveal the best approach to meet the user's needs. At some point, a library may become quite large and cumbersome, and we hope the tools provided in this package allow users to reorganize **amModelLib** objects as needs change.

## AMModels Shiny app

Many natural resource practitioners are time-crunched and may not work with R on a daily basis, facilitating forgetfulness of R workflow and function names. This should not hamper efforts to codify knowledge in the form of models. To that end, we introduce a Shiny application called **AM Model Manager**. To use the application, you must have the shiny and shinyBS packages installed. You can then launch the application with the function modelMgr with no arguments. When the application is launched, the R console will be "listening" and unavailable for use.

*# launch the Model Manager*

**modelMgr**()

The app enables a user to add, delete, and send **amModel** and **amData** objects from the library to R's global environment, and to work with their associated metadata. Once those tasks are completed, the Model Manager is closed and the R console is then freed for additional work.

**Fig 6:**
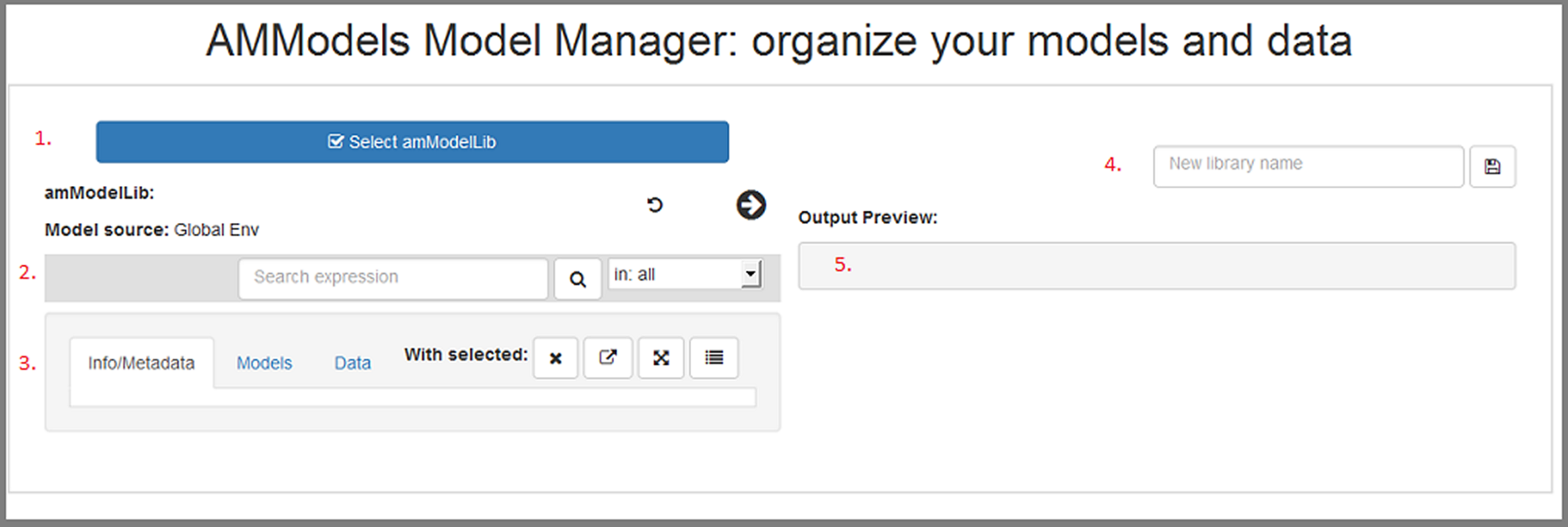
The AMModels Model Manager application main window.

The Shiny application can be resized as desired, and is divided into 5 main actions, highlighted in red in Fig 6:

1. Locate, upload, or create an **amModelLib** object.
2. Search or subset the loaded **amModelLib**.
3. Edit a selected **amModelLib** content by adding or removing metadata, models, or data.
4. Bind an **amModelLib** object to R's global environment, where it can be saved with the **save** or **saveRDS** functions within R's console.
5. View the result of actions taken.

Each of these will be briefly demonstrated.

### Selecting an amModelLib

**Fig 7:**
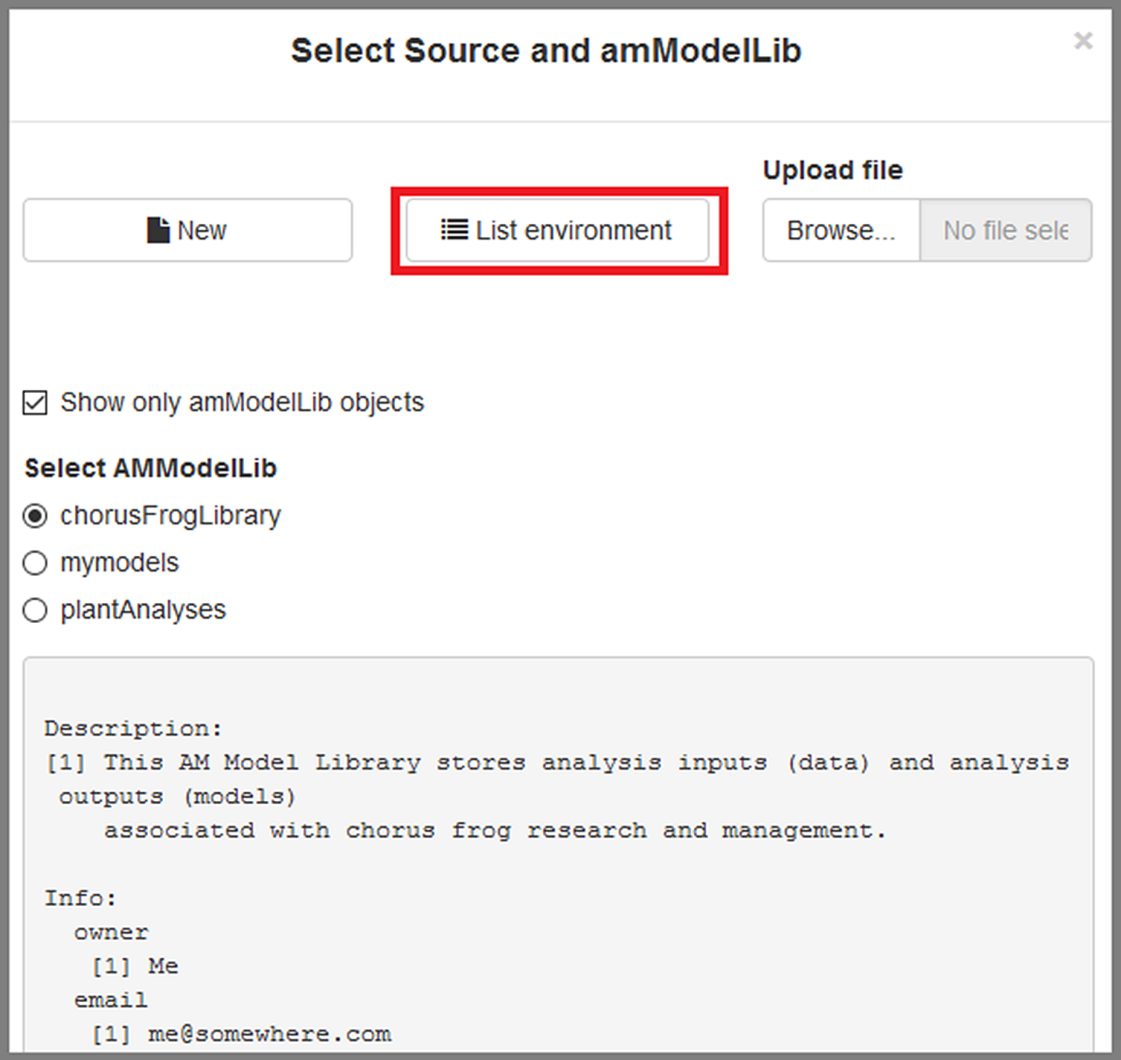
The "Select amModelLib" window allows users to select a library that exists in R's global environment.

Use the blue button at the top of Fig 6 to (1) locate an **amModelLib** that is present in the global environment, (2) create a new library, or (3) upload an .RData, .rda, or .RDS file containing one. When the window first opens, the app will list the objects in your global environment, with the filter to display only **amModelLib** objects active (Fig 7). An **amModelLib** object may be selected from the list displayed if desired. In Fig 7, the **chorusFrogLibrary** has been selected.

**Fig 8:**
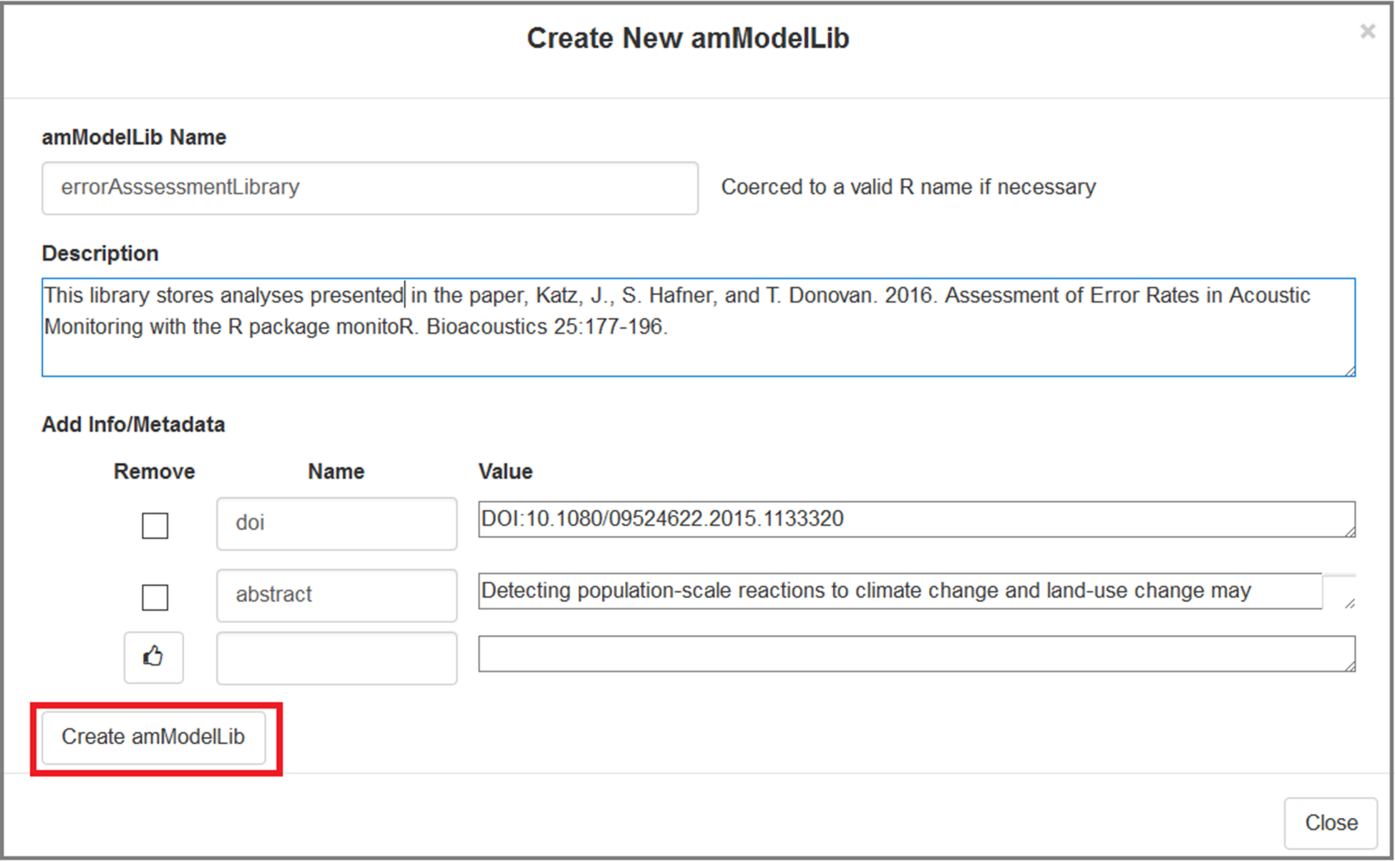
The "Create New amModelLib" window allows users to create a new library.

Alternatively, an **amModelLib** can be created by pressing the "New" button in the app (Fig 7). Creating a new **amModelLib** launches another dialogue box on top of the first one where the user can specify the name of the new **amModelLib**, provide a description, and begin entering metadata associated with the library itself (e.g., Fig 8). The name must be a valid R object name that does not start with a number or contain a space. If these are ignored, the name will be coerced to a valid name using the make.names functions. The "Create amModelLib" button at the bottom of the dialogue box adds the **amModelLib** object to the global environment, loads it as the **amModelLib** chosen for editing in the Model Manager app, and closes the dialogue box. To cancel, choose "Close" at the lower right, or click anywhere outside of the dialogue box.

**Fig 9:**
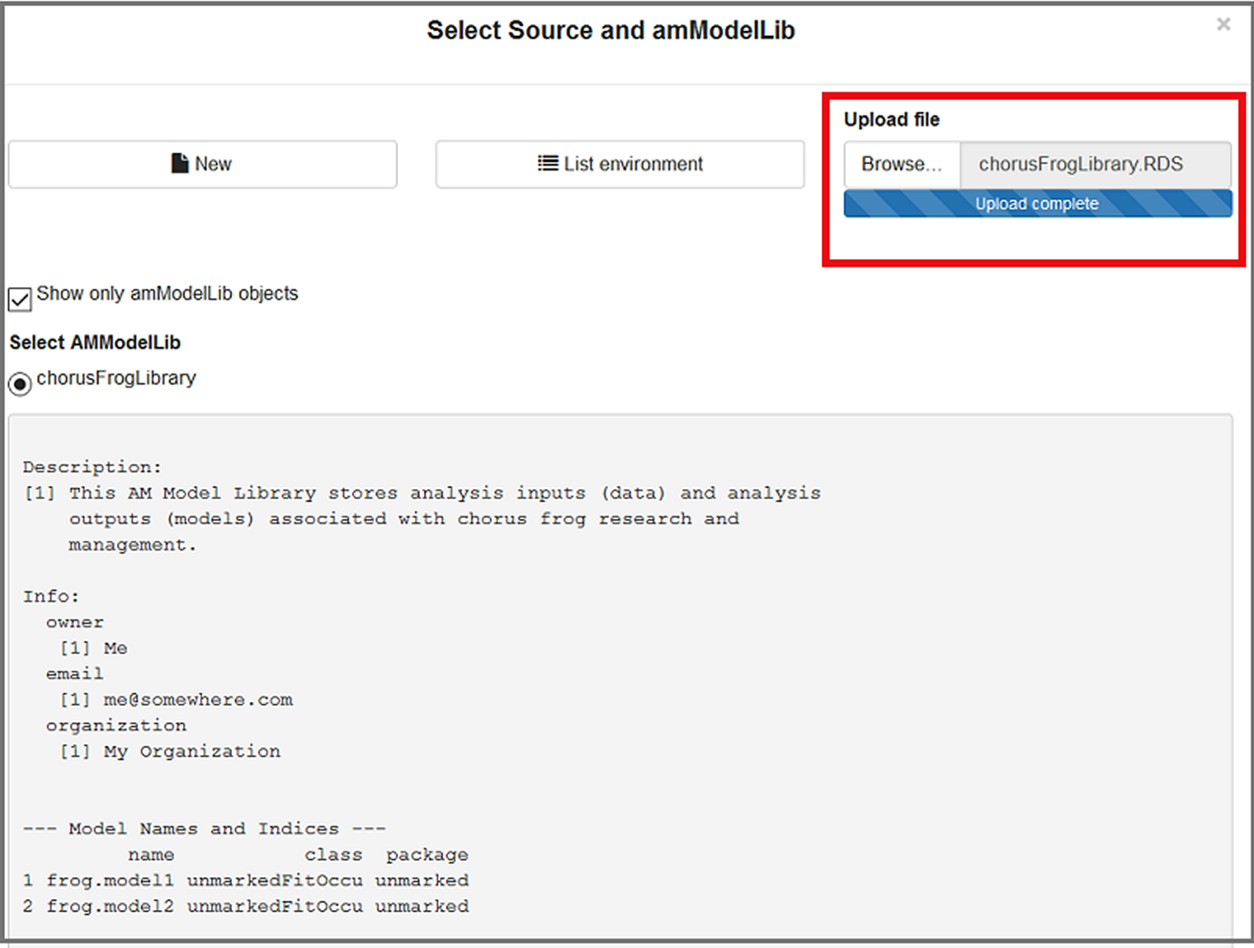
The "Upload file" window allows users to browse to an existing library that is stored as an.RDS or.rda file.

Finally, a user may press the Browse button to browse to an existing .RData or.rda file containing one or more **amModelLib** objects, or an .RDS file containing a single **amModelLib** object (Fig 9). For .RData and .rda files, all objects within the file are loaded into the global environment and displayed as a list in the dialogue box where the user may select one. For .RDS files, the **amModelLib** object is loaded into the global environment using the basename of the uploaded file, minus the .RDS extension. In Fig 9, we browse to the **amModelLib** called **chorusFrogLibrary.RDS** that was previously created in this vignette.

### Editing contents of a loaded library

**Fig 10:**
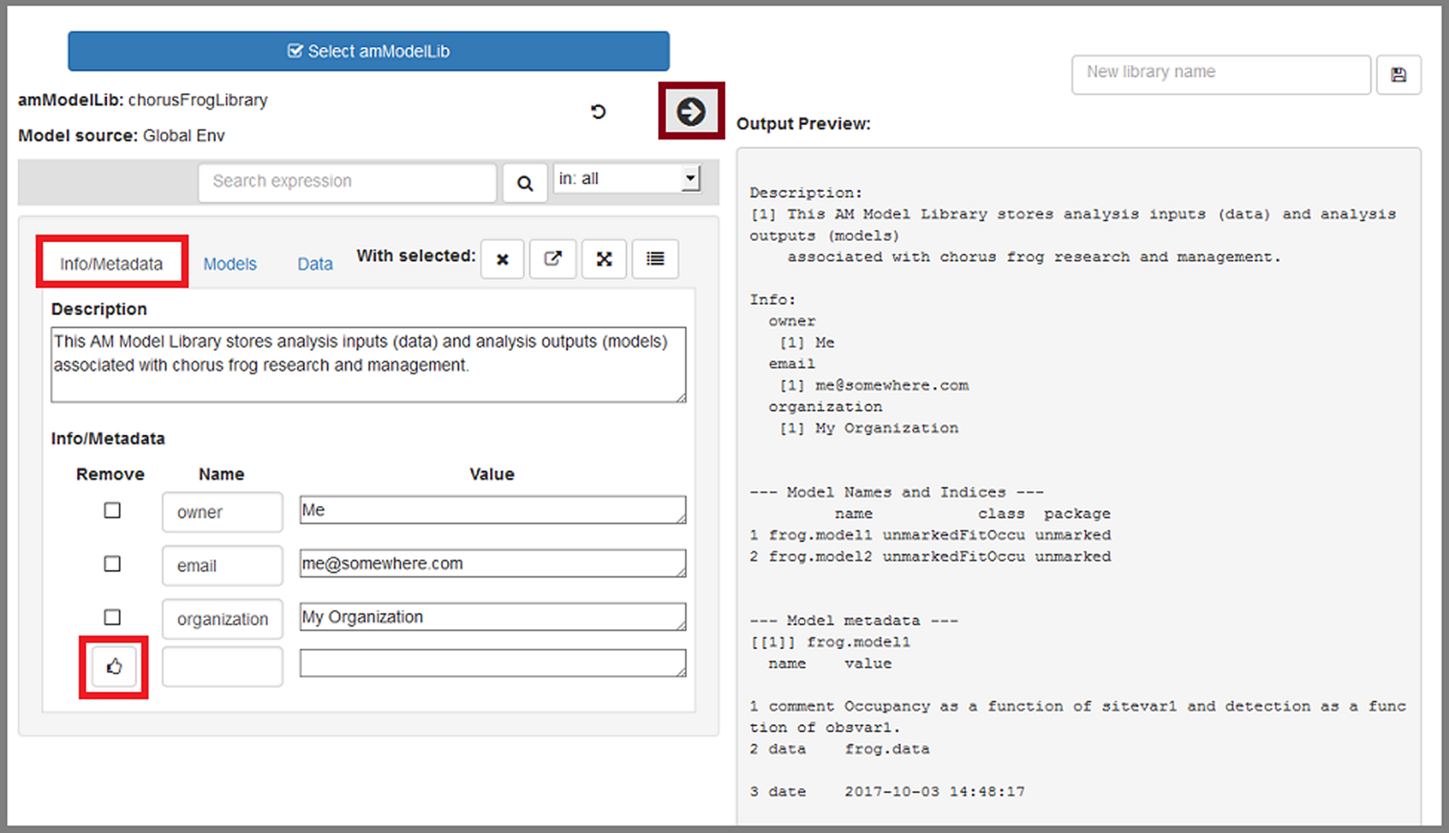
The "Info/Metadata" tab allows users to work with the library's metadata and info slots.

**All edits are contained in the app itself; ultimately any edits will need to be saved as a new object (library) to R's global environment, where they can be saved to file when the app has been closed.** Pressing the right-pointing arrow that is highlighted in red will display a summary of current contents of the loaded library (Fig 10). Here, we discuss how edits within the app are made.

#### Info/Metadata tab

The info/metadata tab contains information about the **amModelLib** itself (Fig 10). The description field is a place to store notes about the contents of the **amModelLib**, while the metadata consist of name-value pairs. Each name-value pair is entered by typing a name for each item and its corresponding value. The values are text fields of any length. After entering a name-value pair, press the "thumbs-up" button to the left of the row to add the item and create an empty row at the bottom of the table, where another pair may be added. To remove a name- value pair, check the box to the left of the row and press the thumbs-up button.

#### Models tab

**Fig 11:**
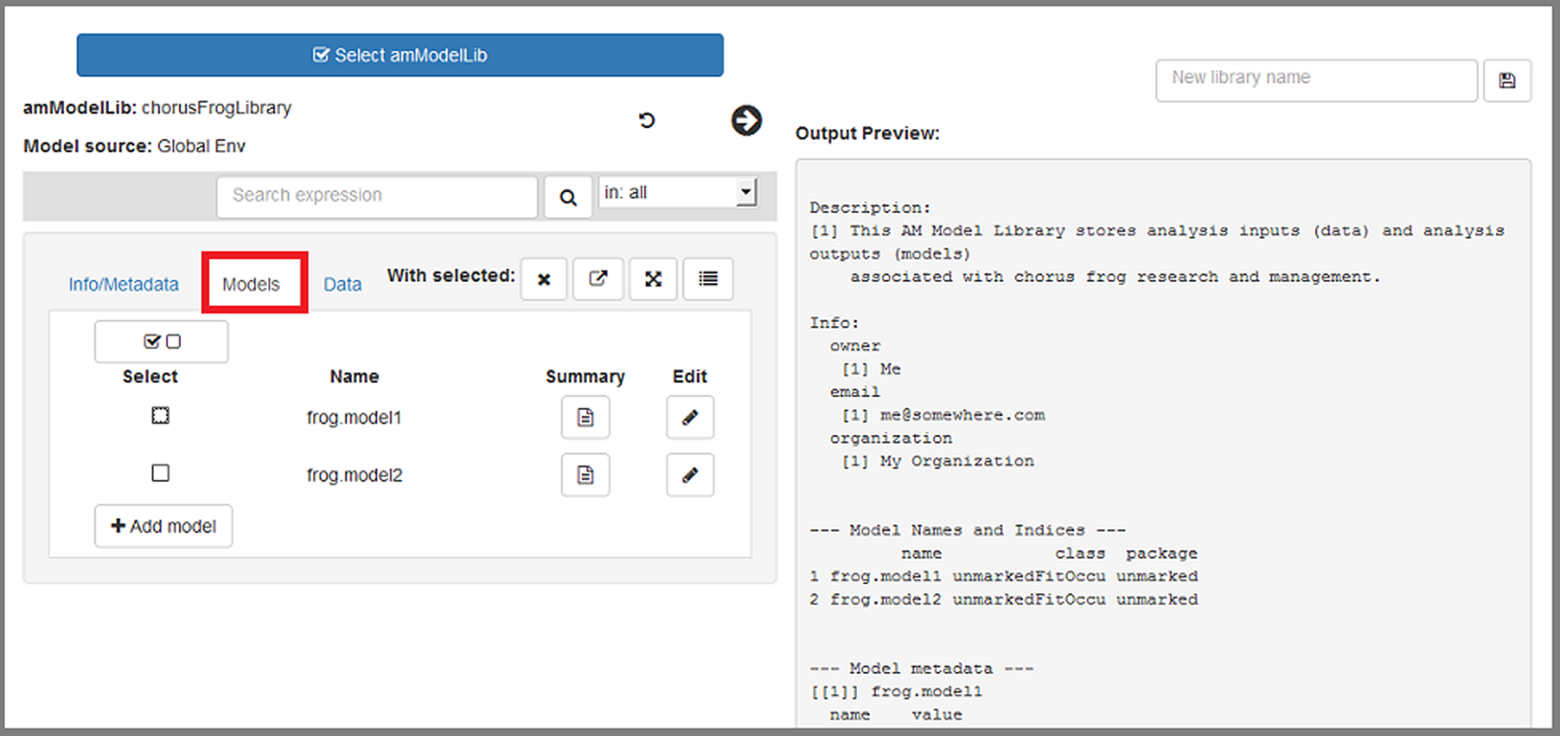
The "Models" tab allows users to add new models, delete existing models, or send models to R's global environment.

The models tab lists the models (analysis outputs) in the **amModelLib** (Fig 11). The "Summary" button in each row opens a dialogue box that displays a summary of the model in that row, invoking the summary method of the object's class. Similarly, the "Edit" button in each row opens a dialogue box that allows the user to edit the metadata associated with the model of that row.

Additional models may be added by clicking the "Add model" button, which opens a dialogue box in which objects from the global environment may be selected for inclusion. The action will convert the object into an **amModel** object and add it to the library. Once the new model has been added, the user can return to the models tab and may press the "Edit" button to supply metadata.

One or more models may be selected by checking the box to the left of the name. Alternatively, all models may be selected or de-selected by pressing the button above the word "Select." Four actions may be taken with selected items, which will be described shortly.

#### Data tab

**Fig 12:**
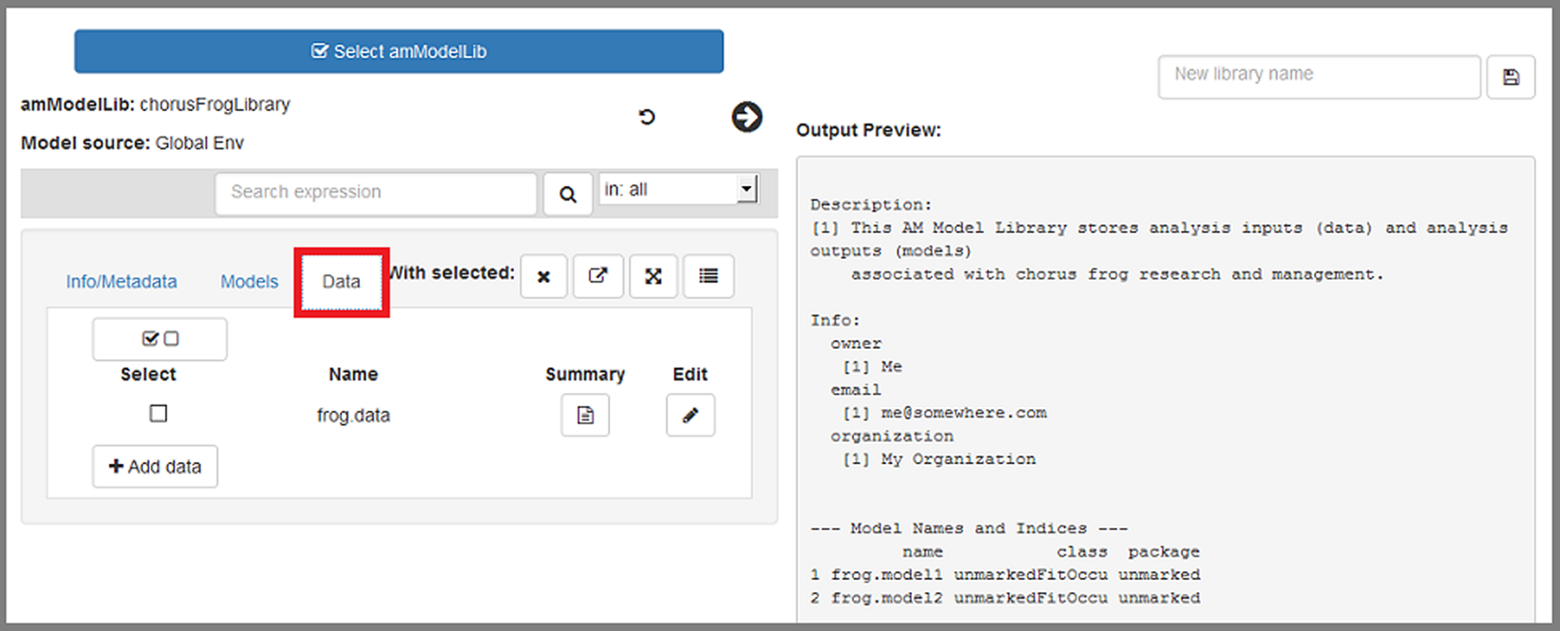
The "Data" tab allows users to add new datasets, delete existing datasets, or send datasets to R's global environment.

The data tab lists the data in the **amModelLib**. Layout and function of the data tab is identical to the models tab (Fig 12).

#### Actions available for selected components

**Fig 13:**
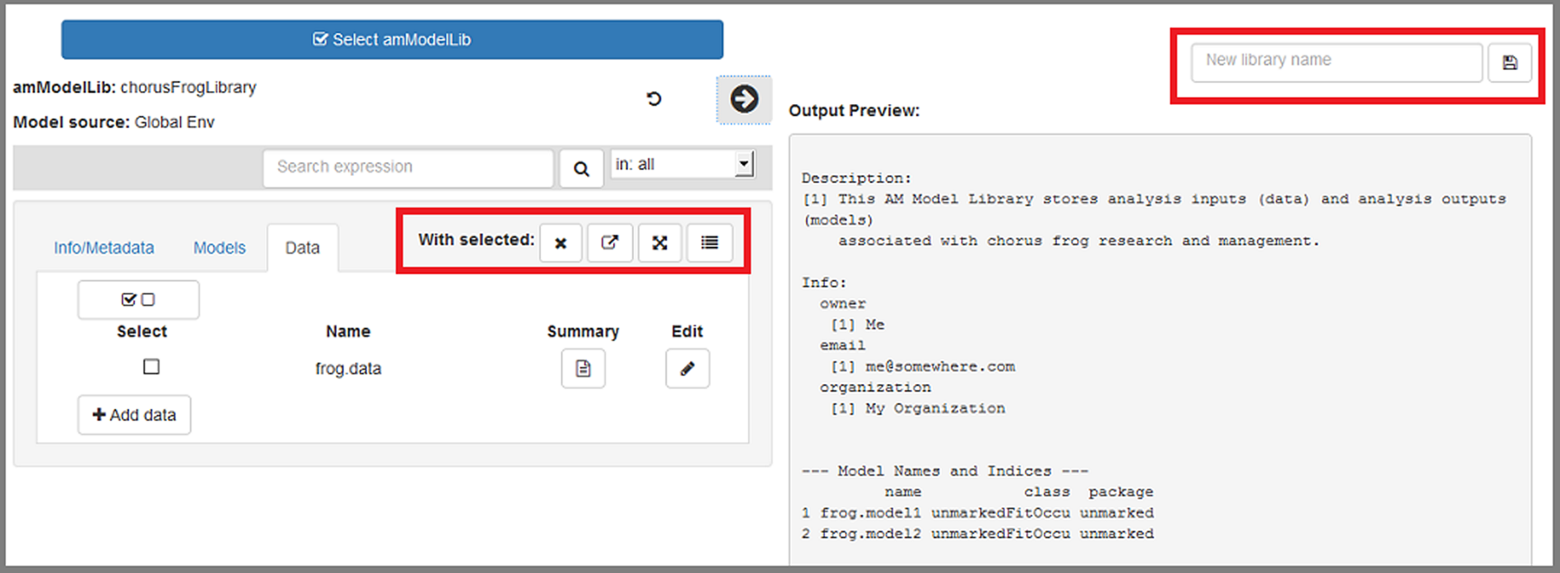
Models and data that are selected can be deleted, subset to a new library, or sent to R's global environment in its original form or as a list.

Four actions can be performed with selected components (Fig 13).

When an action is chosen it is performed on all selected components, both models and data, even though only one of the models or data tab can be visible at a time. Actions are selected using the buttons to the right of the tab-selection bar.

1. Delete. The "delete" action removes the selected components from the loaded **amModelLib** object.
2. Create new **amModelLib**. This action sets the selected objects as a new, unsaved **amModelLib** object. Pressing the right arrow button allows the user to see a summary of this new library.
3. Extract ("check out") an original object from the library to the global environment without metadata (uses **getAMModel** or **getAMData**)
4. Extract ("check out") as a list the original object plus the metadata to the global environment (uses **getAMModel** or **getAMData** with the argument as.list = TRUE). This extraction action, and the one above, immediately place the selected objects into the global environment. Each object placed in the global environment is assigned the same name as in the **amModelLib** object, replacing existing objects with the same name without warning.

For the first two actions, pressing the right-arrow button will show a summary of the resulting **amModelLib**. (Extracting objects will not change the library.)

To undo all actions since the **amModelLib** was loaded, e.g. to undo accidentally deleting some components, click the "revert" button above the search bar (the counter-clockwise arrow).

### Searching for content

**Fig 14:**
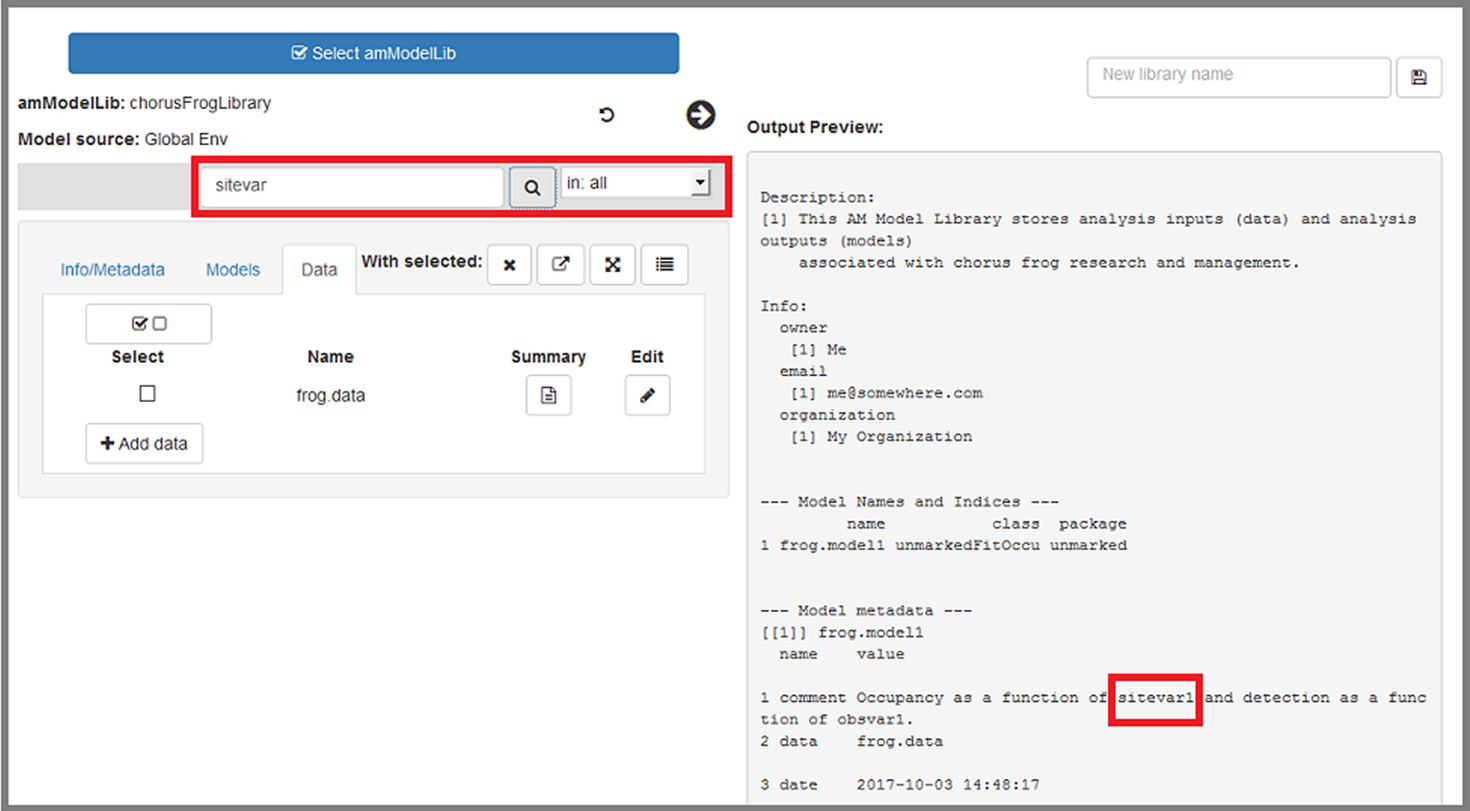
The "Search" panel allows users to search a library by term. Pressing the right arrow will display the result, which can be saved as a new library by providing a name in the New object name box and pressing the Save button, which will bind this new library to R's global environment, where the library can be saved with the save or saveRDS function.

The search box allows users to search the models or datasets in the **amModelLib** using the grepAMModelLib function. Enter a search term or expression in the box, choose whether to search all models and data, just models, or just data, and click the magnifying glass button (Fig 14). A summary of the result of the search is displayed in the output preview pane when the right arrow button is pressed. The results are contained in a new **amModelLib** within the app itself.

### Saving changes

All edits are contained in the app itself; ultimately any edits will need to be assigned to a new object (library) and moved to R's global environment, from where it can be saved to a file using either the save or saveRDS functions in the R console.

To save **all** edits to the loaded library, press the right-arrow to preview the updated contents of the loaded library. Next, supply a "New library name" at the top of the output preview pane and press the "bind" button (with a disk icon which looks like a save button). This action places the library in R's global environment. If the name provided currently exists in the global environment, the original library will be replaced with the updated library. If this action is successfully executed, a success indicator will flash on the screen. Then, once the app is closed, the library within the global environment can be saved with the save or saveRDS functions.

To create a **new** library that is a subset of objects in the loaded library, select the models and data of interest, preview the results of the library subset by pressing the right arrow button, then provide a unique "New library name" and press the "bind" button. If this action is successfully executed, a success indicator will flash on the screen. Once the library is placed in the global environment, the app can be closed and the new **amModelLib** object can be saved to a file using either the save or saveRDS functions. Be cautious of maintaining items that are stored in multiple libraries.

## Versioning

AMModels was developed under R version 3.2.2. We made an attempt to eliminate unnecessary dependencies and imports to minimize the chance that a dependent package update will break functions in this package. The shiny user interface was developed under shiny 1.0.0; this interface is completely dependent on the shiny package, and we expect that we will need to update the shiny UI regularly due to the fast pace of shiny package development. Since the premise of our package is long-term storage, we intend to retain backwards compatibility in our updates to the greatest extent possible, short of asking users to stop updating R. We cannot predict which datasets or analytical routines may be rendered incompatible with a new major version of R or a new package version, so we recommend users note R and package versions in their metadata to retain the information necessary to reproduce an analysis long into the future.

## Summary

Hallgren and Westberg [33] note, "Behind [the theory of AM] lies a strong, albeit implicit, expectation that organizations aiming for AM have the capacity to communicate in a way that facilitates the required coordination of the knowledge perspectives involved." Thus, at an agency or organizational level, adaptive management requires knowledge management—defined as the process of capturing, developing, sharing, and effectively using organizational knowledge to fulfill its mission [34]. Models are a main ingredient of adaptive management programs, and are a formal representation of knowledge. They play a key role in representing uncertainty, can be compared with competing models, and can be used to predict the outcome of a given management action [35]. The R package, **AMModels**, is a flexible and simple package intended to facilitate adaptive management efforts by storing models, along with datasets, so that they can be used to aid in decision making. Although an adaptive management framework includes many other critical ingredients [11,36,37], **AMModels** may provide a useful tool in advancing the use of adaptive management.

The utility of **AMModels**, however, may be useful to those not engaged in an adaptive management setting. Preserving data, outputs, and metadata (that may include the analytical code) can promote reproducibility of scientific analysis [38–40] and foster the use of models into the future.

## Supporting information

S1 Fig Function cheat sheet for the R package, AMModels. (PDF)

S1 Text R script that used in the AMModels examples. (TXT)
