# Supplementary material for: AMModels: An R package for storing models, data, and metadata to facilitate adaptive management
Source: PLoS One. 2018 Feb 28;13(2):e0188966. doi: 10.1371/journal.pone.0188966 (PMC5830045; doi:10.1371/journal.pone.0188966)

AMModels: An R Package for Storing Models, Data, and Metadata to Facilitate Adaptive Management

AMModels Cheat Sheet

```
# create an amModelLib object
mymodels <- amModelLib(
   description = "This AM Model Library
stores models and data.",
   info = list(owner = "me", email =
"me@somewhere.com"))

# view the amModelLib
mymodels

# summarize the amModelLib
summary(mymodels)

# insert amData to amModelLib
mymodels <- insertAMModelLib(data =
list(plant.data = plant.data), amml =
mymodels)

# insert amModel to amModelLib
mymodels <- insertAMModelLib(models =
list(plant.model = plant.model), amml =
mymodels)

# search for data containing the word
'plant'
getAMModelLib(pattern = "plant", amml =
mymodels, search = "data")

# create new amModelLib that includes the
plant and frog models
mymodels2 <- mymodels[c('plant.model',
'frog.model1', 'frog.model2')]

# save the amModelLib
save(mymodels, file = "mymodels.rda")

# load an existing amModelLib
load("mymodels.rda")
```

```
# show the amModelLib description
ammlDesc(mymodels)

# update the description
ammlDesc(mymodels) <- "This AM
Model Library stores models and data
associated with the AMModels package
vignette."
```

```
# look at the metadata associated with the amModelLib
ammlInfo(amml = mymodels)

# extract only the owner
ammlInfo(amml = mymodels, "owner")

# deletions are done by setting the value associated with a key to NULL
ammlInfo(mymodels) <- list(date.created = NULL)

# update the owner name, and add a new metadata element
ammlInfo(mymodels) <- list(owner = 'Me', organization = 'My Organization')
```

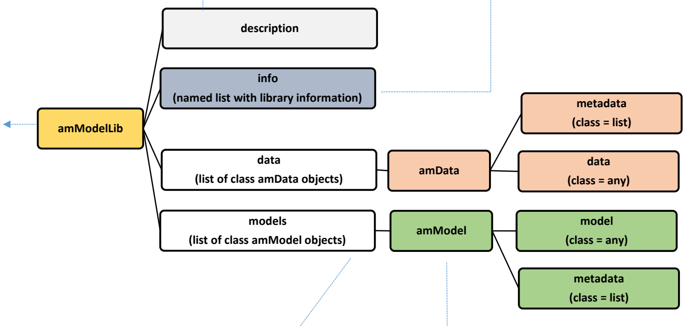


```
# create an object of class amData, which includes the dataset
# and corresponding metadata.
plant.data <- amData(
   data = plant.wt,
   comment = 'Plant dataset from the lm helpfile.',
   taxa = 'plants'


# view summary of an amData object
summary(plant.data)

# insert the data to the amModelLib
mymodels <- insertAMModelLib(models = list(plant.model = plant.model),
amml = mymodels)

# look at names of the existing datasets
lsData(mymodels)

# view all amData metadata
dataMeta(amml = mymodels)

# view metadata associated with a specific amData object
dataMeta(amml = mymodels, x = "plant.data")

# add additional metadata to an amData object in the form of a list
dataMeta(mymodels, 'plant.data') <- list(
   url = "https://stat.ethz.ch/R-manual/R-devel/library/stats/html/lm.html")

# extract and amData object from the library and return dataset to the
global environment
extracted.plant.model <- getAMModel("plant.model", amml = mymodels,
as.list = FALSE)

# extract and amData object from the library and return dataset and
metadata to the global environment as a list
extracted.plant.model <- getAMModel("plant.model", amml = mymodels,
as.list = TRUE)

# remove an amData object from the library
mymodels <- rmData('plant.data', amml = mymodels)
```

```
# create an amModel and add metadata
# notice the element named data can connect the amData object used
# to create the model
plant.model <- amModel(
   model = lm.D9,
   comment = 'Analysis from lm helpfile',
   data = 'plant.data')

# view summary of an amData object
summary(plant.model)

# insert the model to the amModelLib
mymodels <- insertAMModelLib(models = list(plant.model = plant.model), amml =
mymodels)

# look at names of the existing models
lsModels(mymodels)

# view all amModel metadata
modelMeta(amml = mymodels)

# view metadata associated with a specific amModel object
modelMeta(amml = mymodels, x = "plant.model")

# add additional metadata to an amData object in the form of a list
modelMeta(mymodels, 'plant.model') <- list(
   url = "https://stat.ethz.ch/R-manual/R-devel/library/stats/html/lm.html")

 # extract and amModel object from the library and return model to the global
environment
extracted.plant.model <- getAMData("plant.model", amml = mymodels, as.list =
FALSE)

# extract and amModel object from the library and return model and metadata to
the global environment as a list
extracted.plant.model <- getAMModel("plant.model", amml = mymodels, as.list =
TRUE)

# remove the amModel 'plant.model' from the amModelLib
mymodels <- rmModel('plant.model', mymodels)
```

```
# launch Model Manager
modelMgr()
```

AMModels Model Manager: organize your models and data

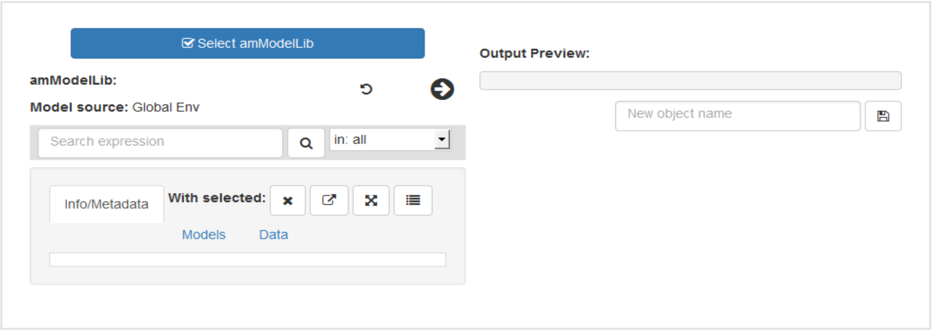

Supplement: S1 Fig — (PDF) [file pone.0188966.s001.pdf]
